# Synthesis of Thermoresponsive Diblock Copolymer Nano-Objects via RAFT Aqueous Emulsion Polymerization of Hydroxybutyl Methacrylate

**DOI:** 10.1021/acs.macromol.2c00379

**Published:** 2022-04-17

**Authors:** Saul J. Hunter, Nicholas J. W. Penfold, Elizabeth R. Jones, Thomas Zinn, Oleksandr O. Mykhaylyk, Steven P. Armes

**Affiliations:** † Dainton Building, Department of Chemistry, University of Sheffield, Brook Hill, Sheffield, Yorkshire S3 7HF, U.K.; ‡ DSM Biomedical, Urmonderbaan 22, 6167RD Geleen, The Netherlands; § ESRF - The European Synchrotron, 38043 Grenoble, France

## Abstract

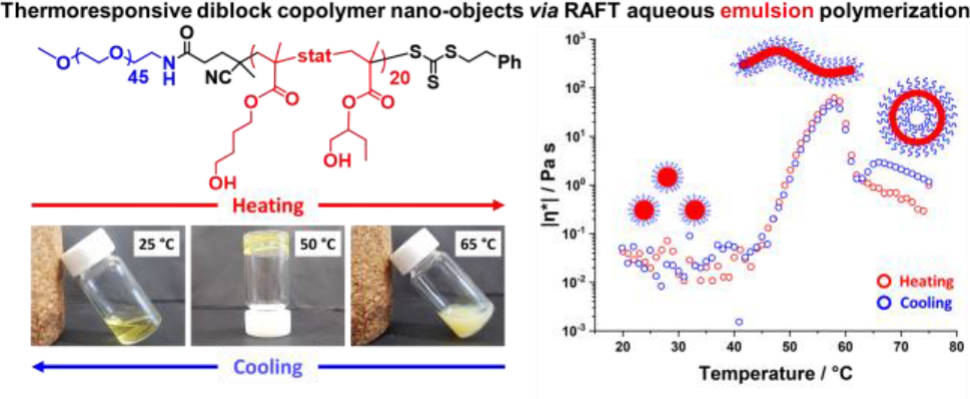

We recently reported that the reversible addition–fragmentation chain transfer (RAFT) aqueous emulsion polymerization of hydroxybutyl methacrylate (HBMA) using a relatively short non-ionic poly(glycerol monomethacrylate) (PGMA) precursor enables convenient preparation of diblock copolymer nano-objects with spherical, worm-like, or vesicular morphologies. We postulated that the relatively high aqueous solubility of HBMA (∼25 g dm^–3^ at 50 °C) was likely to be a key parameter for overcoming the problem of kinetically trapped spheres that is observed for many RAFT aqueous emulsion polymerization formulations. In this study, we revisit the RAFT aqueous emulsion polymerization of HBMA using a poly(ethylene glycol) (PEG) precursor as a steric stabilizer block. Remarkably, the resulting PEG_45_–PHBMA_20_ diblock copolymer nanoparticles exhibit thermoreversible morphological transitions in aqueous solution. More specifically, transmission electron microscopy and small-angle X-ray scattering studies confirmed that spheres are formed at 25 °C, worms at 58 °C, and vesicles at 65 °C. This is the first time that such behavior has been reported for nano-objects prepared by RAFT aqueous emulsion polymerization. Moreover, variable temperature dynamic light scattering and oscillatory rheology studies confirmed that these transitions are highly reversible at 0.1 and 10% w/w, respectively. Variable temperature ^1^H NMR studies indicated that (i) the PEG stabilizer block undergoes dehydration on heating and (ii) the apparent degree of hydration of the hydrophobic PHBMA block increases on heating from 25 to 65 °C. This suggests that the change in copolymer morphology is best explained in terms of a uniform plasticization mechanism.

## Introduction

It is well-known that polymerization-induced self-assembly (PISA) enables the convenient and efficient preparation of a wide range of diblock copolymer nano-objects in the form of concentrated dispersions.^1−26^ In the case of aqueous PISA formulations, if the vinyl monomer used to grow the hydrophobic second block is water-miscible, this corresponds to an aqueous dispersion polymerization.^3,17,21,27−35^ On the other hand, if the monomer is water-immiscible—which is much more common—this corresponds to an aqueous emulsion polymerization.^36−38^ In the PISA literature, there has been substantial interest in the rational design of aqueous dispersions of thermoresponsive shape-shifting diblock copolymer nano-objects over the past decade.^17,39−46^ In practice, such nano-objects are invariably prepared via RAFT aqueous dispersion polymerization.^3,47−50^ This is because water-miscible vinyl monomers such as 2-hydroxypropyl methacrylate (HPMA),^27,51−54^ 4-hydroxybutyl acrylate (HBA),^35,46,55^*N*-isopropylacrylamide,^17,42^ or 2-methoxyethyl acrylate^29,31^ produce homopolymers that are only weakly hydrophobic. In particular, their degree of (partial) hydration is temperature-dependent, which affects the relative volume fraction of such hydrophobic blocks.^39,46^ This induces a subtle change in the fractional packing parameter *P*,^56,57^ which is sufficient to drive various morphological transitions for the corresponding diblock copolymer nano-objects when adjusting the solution temperature.^39,45,46,54,55,62−66^ For example, Ratcliffe and co-workers reported that a single poly(2-hydroxypropyl methacrylamide)–poly(2-hydroxypropyl methacrylate) (PHPMAC_41_–PHPMA_180_) diblock copolymer formed spheres at 4 °C, worms at 22 °C, and vesicles at 50 °C.^45^

Recently, we have reported five examples of HBA-based thermoresponsive diblock copolymers of fixed composition that can form spheres, worms, or vesicles in aqueous solution simply by adjusting the solution temperature.^46,58−61^ Initially, Byard et al. prepared thermoresponsive poly(*N*,*N*′-dimethylacrylamide)–poly(4-hydroxybutyl acrylate) (PDMAC_56_–PHBA_218–269_) worms via RAFT aqueous dispersion polymerization of HBA at 20% w/w solids.^62^ Spheres were formed on cooling to 3 °C, whereas heating to 50 °C led to the formation of vesicles. Variable temperature ^1^H NMR studies indicated that the weakly hydrophobic HBA repeat units became *more* hydrated at elevated temperature. This is in striking contrast to the behavior observed for PHPMA-based diblock copolymers, where the HPMA repeat units become *less* hydrated on heating.^39,53,61,63−67^ Such contrasting behavior is rather counterintuitive given that HPMA and HBA are structural isomers. Unfortunately, the relatively low *T*_g_ of the PHBA block led to film formation, which prevented the determination of copolymer morphologies via TEM.^62^ Subsequently, Byard et al. addressed this technical issue by statistically copolymerizing HBA with a cross-linkable diacetone acrylamide (DAAM) comonomer.^46^ Covalent stabilization using adipic acid dihydrazide prevented film formation and hence enabled TEM studies. However, the presence of 20 mol % DAAM comonomer reduced the thermoresponsive behavior exhibited by the HBA-rich structure-directing block. Recently, this limitation was overcome by Deane et al., who prepared a series of poly(2-(*N*-(acryloyloxy)ethylpyrrolidone)–poly(4-hydroxybutyl acrylate) (PNAEP_85_–PHBA_*x*_) diblock copolymer nano-objects. In this case, glutaraldehyde was reacted with the pendent hydroxyl groups on the PHBA block to covalently stabilize the nanoparticles. This approach enabled high-quality TEM images of PNAEP_85_–PHBA_295_ nano-objects to be obtained without incorporating a second cross-linkable comonomer (e.g. DAAM) within the structure-directing block. This enabled direct evaluation of the thermoresponsive behavior of purely PHBA-based nano-objects. More specifically, raising the temperature drives morphological transitions from spheres (5 °C) to worms (23 °C) to vesicles (31 °C) and finally lamellae (41 °C). Moreover, such transitions proved to be fully reversible on cooling.

In striking contrast, the RAFT aqueous emulsion polymerization of relatively hydrophobic vinyl monomers such as styrene,^68−74^ methyl methacrylate,^75−77^ benzyl methacrylate,^78,79^*n*-butyl acrylate,^73,75,80^ phenyl acrylate,^81^ vinyl acetate,^82−85^ or 2,2,2-trifluoroethyl methacrylate^86−89^ invariably leads to the formation of block copolymer nano-objects that do *not* exhibit any thermoresponsive behavior. Moreover, such formulations often lead solely to kinetically trapped spheres, although there are a few well-known counter-examples to this morphological limitation.^20,69,70,90−94^ Recently, Armes and co-workers explored the RAFT aqueous emulsion polymerization of vinyl monomers such as glycidyl methacrylate,^95−97^ 2-methoxyethyl methacrylate,^98^ or hydroxybutyl methacrylate (HBMA)^99,100^ which exhibit moderate aqueous solubilities (e.g., 15–20 g dm^–3^) at 20 °C. This has provided access to well-defined worms and vesicles, as well as spheres. However, such nano-objects typically do not exhibit thermoresponsive behavior.

Herein we report the RAFT aqueous emulsion polymerization of HBMA using a trithiocarbonate-capped PEG precursor. We demonstrate that this system provides the first example of thermoresponsive diblock copolymer nano-objects to be prepared by using such an aqueous PISA formulation. Moreover, PEG–PHBMA spheres, worms or vesicles can be formed reversibly in aqueous solution simply by varying the solution temperature.

## Results and Discussion

**Figure 1:**
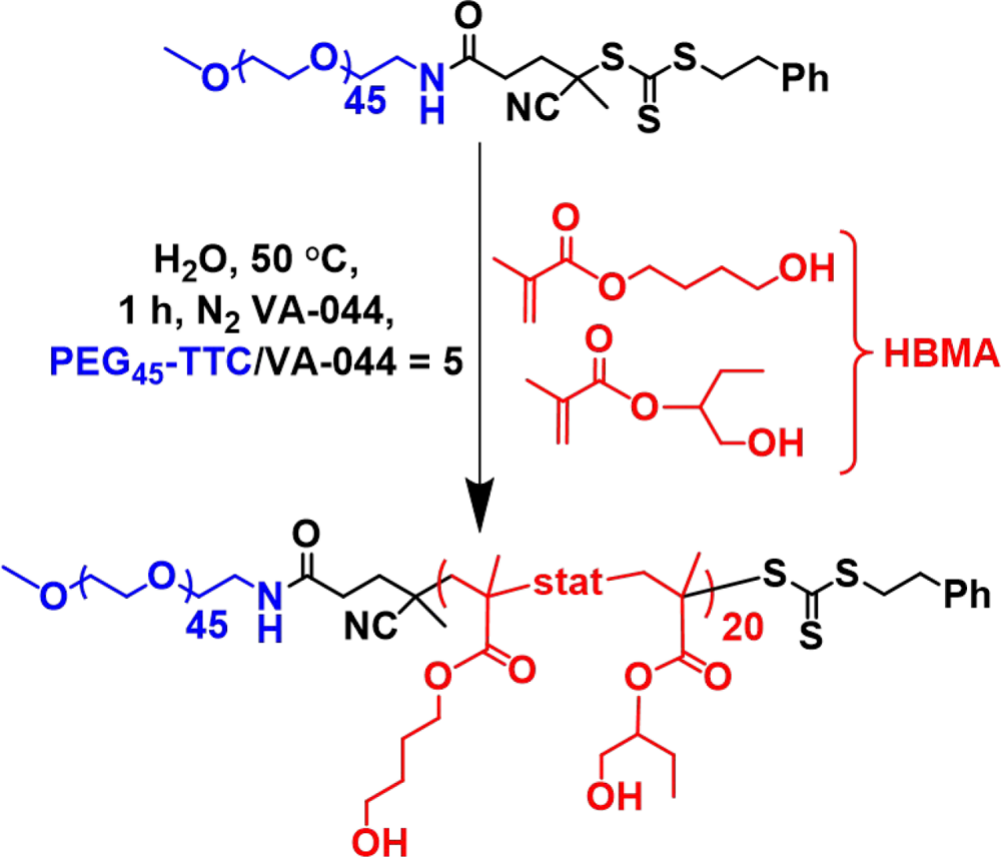
RAFT aqueous emulsion polymerization of HBMA at 50 °C using a trithiocarbonate-based PEG_45_-TTC precursor and a PEG_45_-TTC/VA-044 molar ratio of 5.0 while targeting 10% w/w solids (N.B. HBMA monomer comprises a 1:1 mixture of the 2- and 4-isomers; the chemical structure of both isomers is indicated on the reaction arrow).

PEG_45_–PHBMA_20_ diblock copolymer nano-objects were synthesized by RAFT aqueous emulsion polymerization of HBMA using a previously reported trithiocarbonate-based PEG_45_-TTC precursor^64^ (see Figure 1). These syntheses were performed using an azo-based VA-044 initiator at 50 °C and a PEG_45_-TTC/VA-044 molar ratio of 5.0 while targeting 10% w/w solids.

^1^H NMR spectroscopy studies of PEG_45_–PHBMA_20_ in CD_3_OD (which leads to molecular dissolution of the initial nano-objects) confirmed that a HBMA conversion of more than 99% was achieved within 1 h at 50 °C (see Figure S1). DMF GPC curves obtained for the PEG_45_–PHBMA_20_ diblock copolymer using either a refractive index or a UV detector tuned to the absorption wavelength of the trithiocarbonate RAFT chain-end (λ = 305 nm) are shown in Figure S2. In each case, these GPC curves are shifted relative to that of the corresponding PEG_45_-TTC precursor, which indicates a relatively high blocking efficiency and minimal homopolymer contamination. Moreover, a relatively narrow unimodal molecular weight distribution was obtained (*M*_w_/*M*_n_ = 1.11), which is consistent with those previously reported for PGMA_41_–PHBMA diblock copolymers.^100^ Targeting higher PHBMA DPs led to a systematic increase in *M*_w_/*M*_n_ because of the presence of dimethacrylate impurities in the HBMA monomer, which inevitably causes chain branching.^22,45,101^ Visual inspection confirmed that the final 10% w/w aqueous dispersion of PEG_45_-PHBMA_20_ nano-objects was a transparent free-flowing fluid at 20 °C. This was not unexpected because the relatively short PHBMA DP should favor the formation of spheres. Indeed, TEM studies confirmed that these nano-objects possessed a spherical morphology (see Figure 3a) while DLS studies indicated a *z*-average diameter of 17 nm (PDI = 0.09).

**Figure 2:**
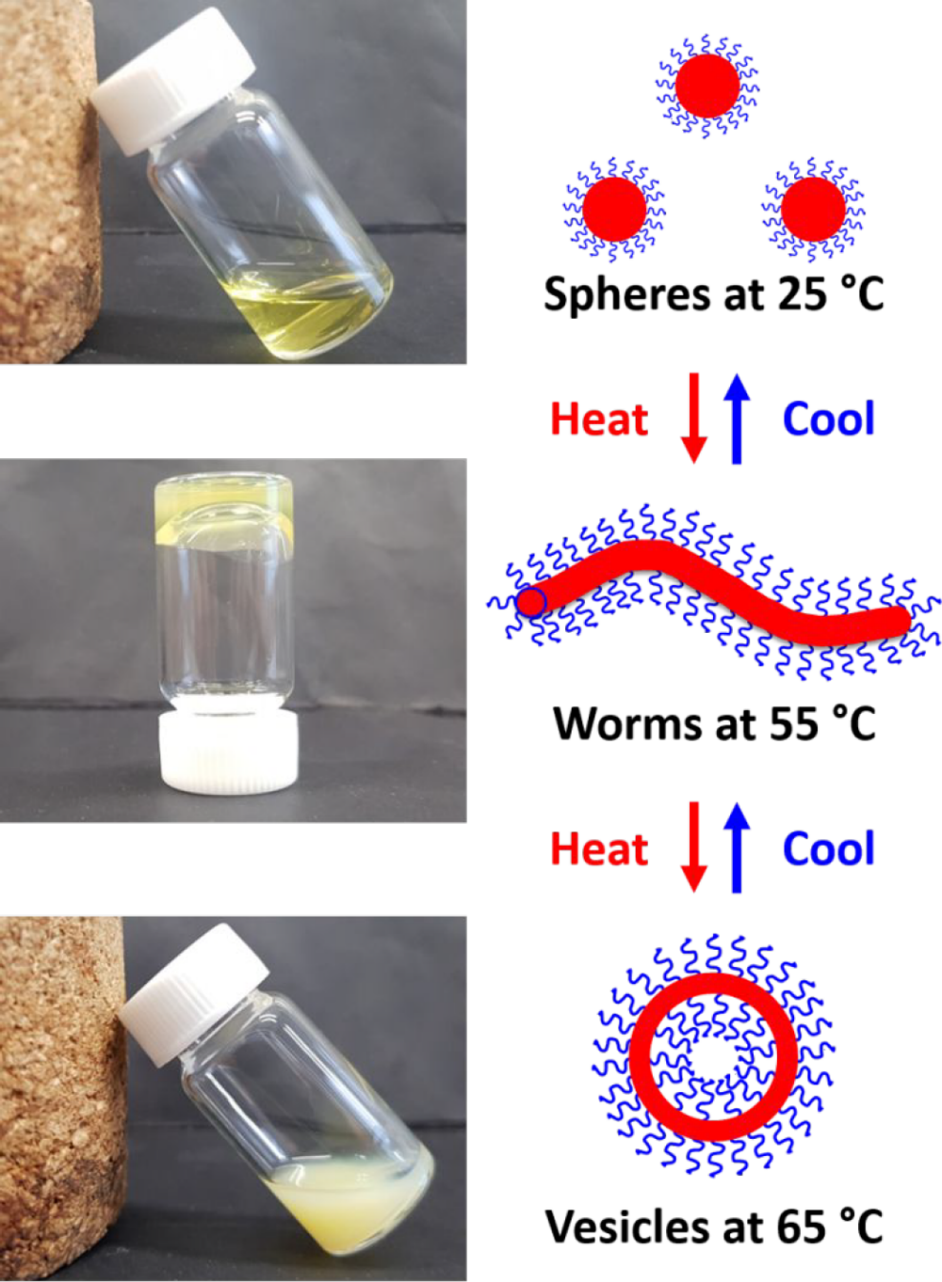
Digital images (left) illustrating the physical appearance of a 10% w/w PEG_45_–PHBMA_20_ aqueous dispersion: (top) at 25 °C, (middle) on heating to 55 °C for 10 min, and (bottom) on heating to 65 °C for 10 min. Schematic representation (right) of the likely thermoreversible morphological transitions exhibited by these diblock copolymer nano-objects. N.B. The observed yellow color arises from the trithiocarbonate chain-ends.

On quenching the HBMA polymerization at 50 °C, the 10% w/w PEG_45_–PHBMA_20_ dispersion was highly viscous. However, a free-flowing dispersion was obtained after cooling to 20 °C. This physical transformation suggested that these PEG_45_–PHBMA_20_ nano-objects were likely to possess thermoresponsive character. To explore this hypothesis, the 10% w/w dispersion was immersed in an oil bath and heated to 25, 55, or 65 °C for 10 min while recording digital photographs (see Figure 2). On heating to 55 °C, a relatively transparent free-standing gel was obtained. On further heating to 65 °C, degelation occurred to produce a free-flowing turbid dispersion. Such transformations proved to be fully reversible as judged by visual inspection.

**Figure 3:**
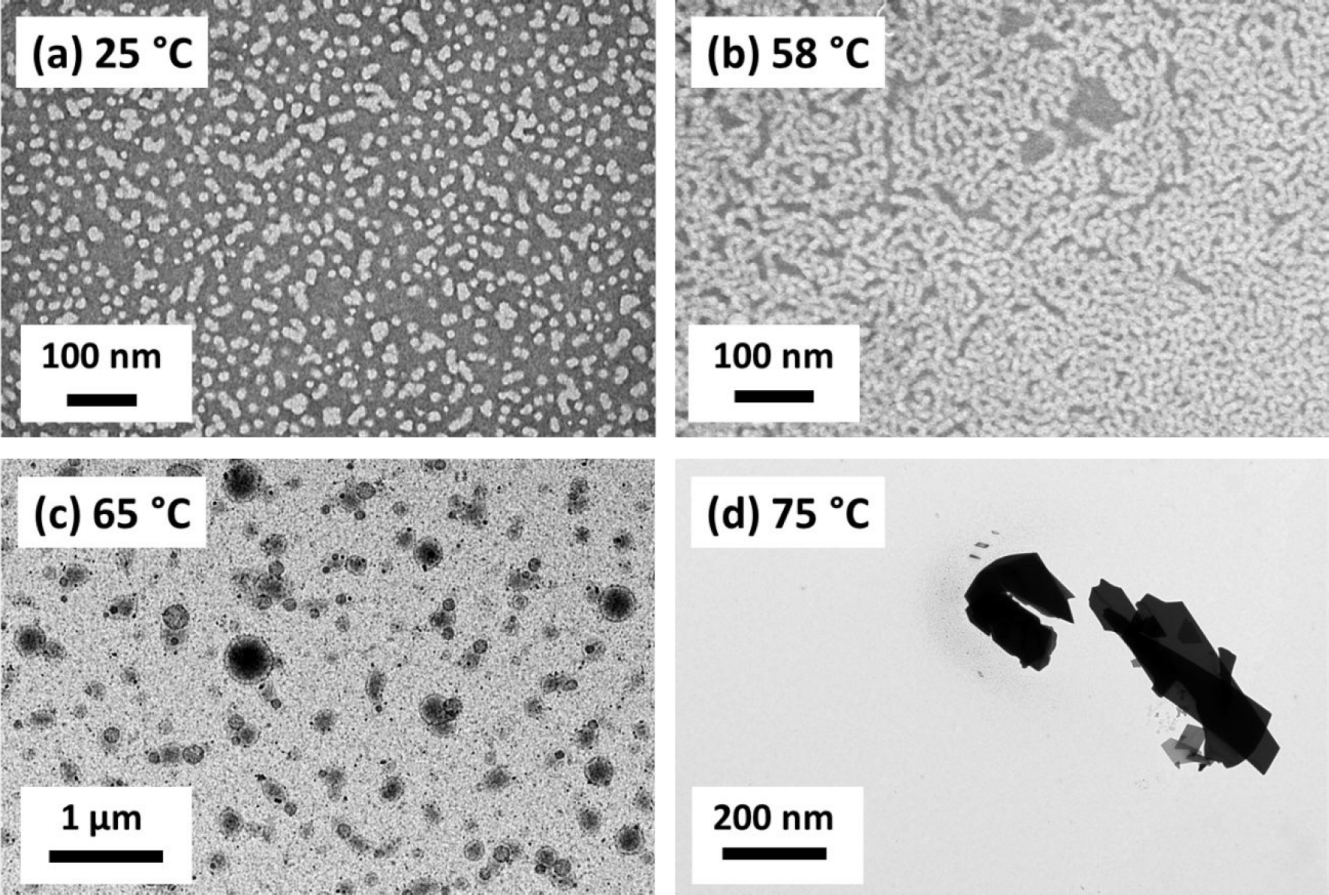
Representative TEM images obtained for a 0.1% w/w aqueous dispersion of PEG_45_–PHBMA_20_ nano-objects at (a) 25 °C (spheres) and after heating for 30 min to (b) 58 °C (worms), (c) 65 °C (vesicles), or (d) 75 °C (lamellae).

There are numerous examples of thermoresponsive diblock copolymer nano-objects in the PISA literature.^33,39,44−46,53−55,59,63,66,102−110^ To examine whether PEG_45_–PHBMA_20_ nano-objects also exhibit thermally-induced changes in morphology, TEM studies were performed for 0.1% w/w aqueous dispersions after drying at 25, 58, 65, or 75 °C. Unlike previously reported temperature-dependent studies on PHBA-based diblock copolymers,^46,59,111^ the PEG_45_–PHBMA_20_ nano-objects studied herein do not require covalent stabilization prior to their visualization by TEM. This is because the methacrylic block has a sufficiently high *T*_g_ to prevent film formation during TEM grid preparation. Hence the PEG_45_–PHBMA_20_ nano-objects can be analyzed directly.^55^ TEM studies confirmed that PEG_45_–PHBMA_20_ undergoes morphological transitions that resemble those reported for PHBA-based diblock copolymers.^112^ More specifically, PEG_45_–PHBMA_20_ forms spheres at 25 °C, anisotropic worms at 55 °C, vesicles at 65 °C, and lamellae at 75 °C (see Figure 3). Such thermoresponsive behavior is perhaps surprising given the significantly greater hydrophobic character of HBMA monomer (aqueous solubility ∼30 g dm^–3^ at 20 °C) compared to that of HBA (which is miscible with water in all proportions at 20 °C). One subtle difference between PHBA- and PHBMA-based diblock copolymer nano-objects is the preferred morphology at ambient temperature. For example, Deane et al. reported that PNAEP_85_–PHBA_295_ worms undergo a worm-to-sphere on cooling to 5 °C, whereas on heating to 34 °C they undergo a worm-to-vesicle transition. In contrast, PEG_45_–PHBMA_20_ forms spheres at ambient temperature, which undergo a sphere-to-worm transition at 55 °C followed by a worm-to-vesicle transition on heating to 70 °C. In summary, these two diblock copolymers undergo the same thermal transitions, but higher onset temperatures are required to produce worms and vesicles in the case of PEG_45_–PHBMA_20_.

**Figure 4:**
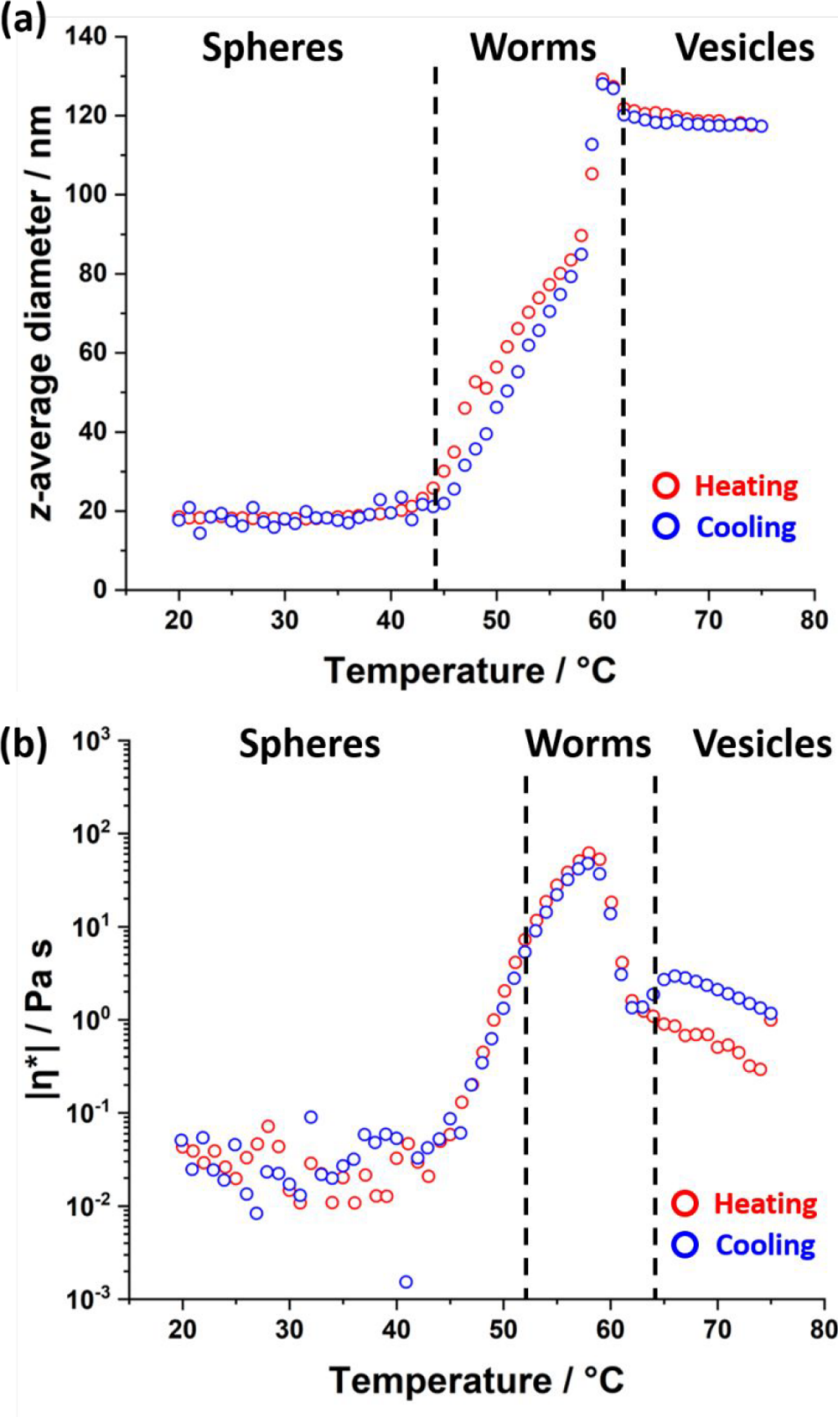
(a) Apparent sphere-equivalent *z*-average diameter determined by DLS as a function of temperature for a 0.1% w/w dispersion of PEG_45_–PHBMA_20_ nano-objects. The red data were recorded on heating from 20 to 75 °C while the blue data were recorded on cooling from 75 to 20 °C. The dispersion was equilibrated at each temperature for 5 min prior to DLS measurements. The black dashed lines indicate the likely phase boundaries for the three copolymer morphologies (spheres, worms, and vesicles). (b) Complex viscosity |η*| as a function of temperature for a 20 °C to 75 °C to 20 °C thermal cycle obtained for a 10% w/w aqueous dispersion of PEG_45_–PHBMA_20_ nano-objects at an applied strain of 1.0% and an angular frequency of 1.0 rad s^–1^. This dispersion was equilibrated at 20 °C for 10 min prior to a thermal cycle conducted at a heating/cooling rate of 1 °C min^–1^. The black dashed lines indicate the sol–gel and gel–sol transitions that occur during this cycle, as indicated from the relationship between *G*′ and *G*″.

DLS was used to determine the sphere-equivalent *z*-average diameter for a 0.10% w/w aqueous dispersion of PEG_45_–PHBMA_20_ nano-objects during a thermal cycle from 20 °C to 75 °C to 20 °C (Figure 4a). Initially, this dispersion comprised relatively small spheres (17 nm diameter, PDI = 0.09). At 44 °C, the *z*-average diameter and DLS polydispersity both begin to increase rapidly, which is characteristic of a sphere-to-worm transition.^46,113^ A dramatic increase in size and a concomitant reduction in DLS polydispersity occur above 60 °C, suggesting the formation of relatively small, well-defined vesicles (*z*-average diameter = 117 nm, DLS polydispersity = 0.05). Comparable *z*-average diameters were recorded during the cooling cycle, indicating remarkably good thermoreversibility at this relatively low copolymer concentration.

Oscillatory rheological studies conducted on a 10% w/w aqueous dispersion of PEG_45_–PHBMA_20_ nano-objects confirmed that a low-viscosity fluid was obtained between 20 and 50 °C (see Figure 4b). Further heating of this dispersion produced a soft, highly transparent, free-standing gel (Figure 2). More specifically, the storage modulus (*G*′) exceeds the loss modulus (*G*″) at around 52 °C (see Figure S3), and a maximum |η*| of 62 Pa s (which corresponds to a maximum *G*′ value of around 45 Pa) is attained at 58 °C (see Figure 4b). According to Lovett and co-workers, such macroscopic gelation is the result of multiple interworm contacts, which leads to the formation of a 3D percolating network.^104^ It is perhaps noteworthy that this PEG_45_–PHBMA_20_ worm gel is somewhat weaker than the PEG_113_–PHPMA_220_ worm gel reported by Warren et al.^28^ (*G*′ = 65 Pa at 11 °C). However, direct comparison between the PEG_45_–PHBMA_20_ worms reported herein and the previously reported PEG_113_–PHPMA_220_ worms is somewhat problematic given the different PEG stabilizer block DPs. Furthermore, a much lower PHBMA DP is required to access the worm phase because this core-forming block is significantly more hydrophobic than either PHBA^58,60,61^ or PHPMA.^27,65^ Heating the PEG_45_–PHBMA_20_ dispersion above 58 °C resulted in a substantial reduction in viscosity, suggesting a worm-to-vesicle transition.^46,114^ Heating to 75 °C led to a second, smaller increase in viscosity, which corresponds to the formation of lamellae (see Figure 3d).^46^ These thermal transitions proved to be remarkably reversible, with relatively little hysteresis being observed at heating/cooling rates of 1 °C min^–1^. Exceptionally, hysteresis is observed for the vesicle-to-lamellae transition: very similar observations were reported by both Wilson^62^ and Deane and co-workers.^58^ This strongly suggests that such hysteresis is most likely characteristic of the precise mechanism for the structural transformation between vesicles and lamellae. Such observations clearly warrant further studies.

Shear-induced polarized light imaging (SIPLI) studies were conducted from 20 to 75 °C to provide further evidence for the thermally induced changes in copolymer morphology. According to Mykhaylyk and co-workers, this optorheological technique enables the alignment of anisotropic nano-objects such as block copolymer worms and lamellae to be visualized at a certain critical rate of applied shear.^115−117^ At 20 °C, only a uniform dark image was observed for the 10% w/w aqueous dispersion of PEG_45_–PHBMA_20_ nano-objects at an applied maximum shear rate of 1.0 s^–1^ (see Figure S4). This lack of birefringence indicates the presence of isotropic spheres. At 60 °C, a distinctive Maltese cross is observed, which is characteristic of anisotropic nano-objects.^65,118^ This feature is the result of birefringence produced by *in situ* shear alignment of the worms.^46^ It disappears above 60 °C, which is consistent with a worm-to-vesicle transition, while a new (albeit weaker) Maltese cross is observed at 70 °C. The latter feature indicates the presence of anisotropic lamellae possessing either a perpendicular or a transverse orientation.^46^ However, the onset temperature required to produce lamellae is lower than the 75 °C indicated by the oscillatory rheology data shown in Figure 4b. This is because the applied (continuous) shear used for the SIPLI experiment produces a greater strain, which promotes the transition from vesicles to lamellae.^46^

**Figure 5:**
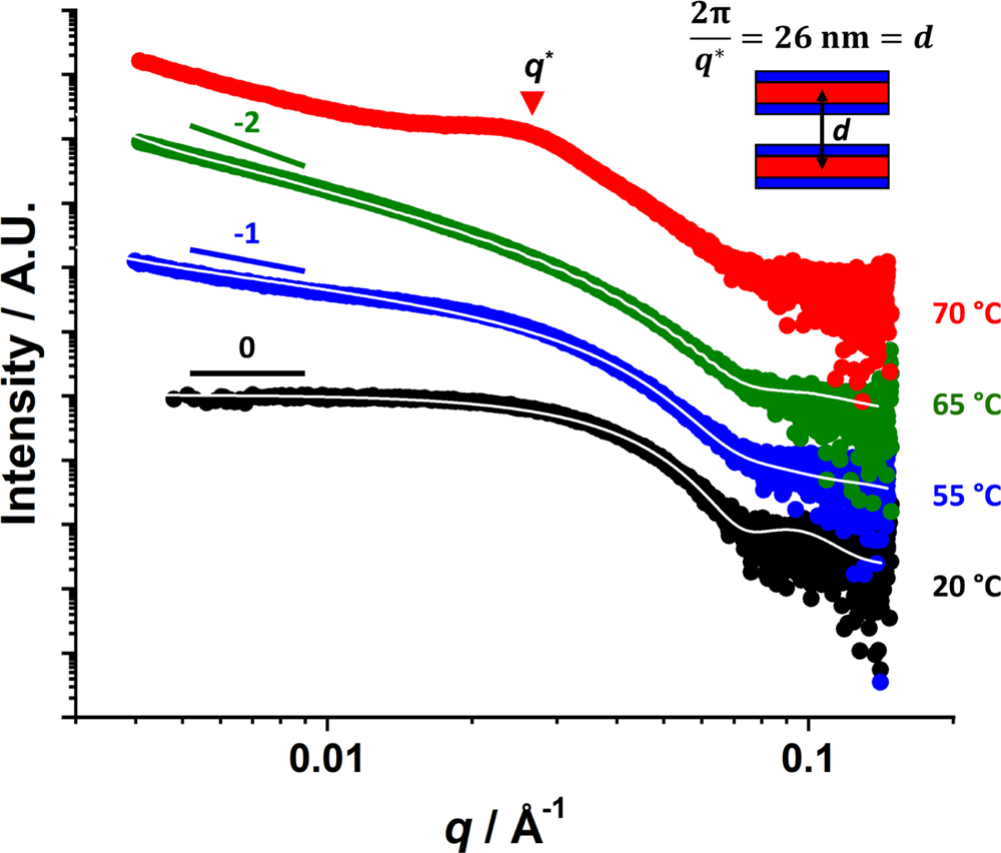
Representative double-logarithmic *I*(*q*) vs *q* SAXS patterns recorded for a 1.0% w/w aqueous dispersion of thermoresponsive PEG_45_–PHBMA_20_ nano-objects at 20 °C (black), 55 °C (blue), 65 °C (green), and 70 °C (red). The solid white line within each of the first three patterns indicates the data fits obtained by using appropriate scattering models (see the Supporting Information for further details).^119,120^ The characteristic low *q* gradients expected for spheres, worms, and vesicles (0, −1 and −2, respectively) are included as a guide for the eye. No data fit could be obtained for the red pattern recorded at 70 °C for the lamellae. However, the mean distance, *d*, between stacked lamellae was calculated from the structure peak labeled q* by using the equation shown in the inset.

Small-angle X-ray scattering (SAXS) studies were conducted on a 1.0% w/w aqueous dispersion of PEG_45_–PHBMA_20_ nano-objects as a function of temperature. Figure 5 shows selected SAXS patterns recorded at 20, 55, 65, and 70 °C; three of these patterns could be satisfactorily fitted (see solid white lines) using well-known scattering models (see the Supporting Information).^104,105^ In each case, the dimensions calculated from these SAXS fits were consistent with those determined by DLS and TEM (see Table 1). SAXS analysis of the spheres formed at 20 °C indicated a volume-average core diameter of 13.8 nm, which is consistent with the overall hydrodynamic *z*-average diameter of 17.6 nm indicated by DLS. For the anisotropic worms formed at 55 °C, the volume-average cross-sectional core diameter *T*_w_ was calculated to be 11.5 nm, which is in reasonably good agreement with the number-average worm width of 11.2 nm estimated by TEM. The gradual increase in the apparent sphere-equivalent DLS diameter at around 40 °C (see Figure 4a) indicates that the formation of these PEG_45_–PHBMA_20_ worms involves stochastic 1D fusion of multiple spheres, which has been observed for other PISA formulations.^22,65,109,113^ In principle, a modest reduction in cross-sectional diameter is expected when cylindrical worms are formed via fusion of multiple spheres (see Figure S5).^54^ Under such circumstances, the worm core diameter divided by the sphere core diameter should be equal to the square root of 2/3, or ≈0.82. This is consistent with the worm/sphere diameter ratio of 11.5 ÷ 13.8 = 0.83 calculated by SAXS analysis of the corresponding PEG_45_–PHBMA_20_ nano-objects.

**Table 1:** Summary of the Various Structural Parameters Calculated from SAXS Analysis of a 1.0% w/w Aqueous Dispersion of PEG_45_-PHBMA_20_ Nano-Objects at 20, 55 or 65 °C^a^

temp/°C	morphology	*D*_s_/nm^b^	*D*_w_/nm^c^	*D*_v_/nm^d^	*T*_m_/nm	*x*_sol_
20	spheres	13.8 ± 2.2				0.001
55	worms		11.5 ± 2.0			0.001
65	vesicles			119 ± 46	10.7 ± 0.6	0.18

a *D*_s_ is the volume-average sphere diameter, *D*_w_ is the volume-average worm cross-sectional diameter, *D*_v_ is the overall volume-average diameter of the vesicles, *T*_m_ is the mean vesicle membrane thickness, and *x*_sol_ is the volume fraction of water within the hydrophobic PHBMA core/membrane.

b Using a spherical micelle model,^120^*D*_s_ was calculated via *D*_s_ = 2*R*_s_ + 4*R*_g_, where *R*_s_ is the mean spherical micelle core radius and *R*_g_ represents the radius of gyration of the PEG_45_ stabilizer block.

c Using a worm-like model,^120^*D*_w_ was calculated via *D*_w_ = 2*R*_w_ + 4*R*_g_, where *R*_w_ is the mean volume-average cross-sectional worm radius.

d Using a vesicle model,^120^*D*_v_ was calculated via *D*_v_ = 2*R*_m_ + *T*_m_ + 4*R*_g_, where *R*_m_ is the mean volume-average radius from the center of the vesicle to the center of the membrane.

At 65 °C, SAXS analysis indicated that relatively small vesicles were formed with a volume-average diameter of 119 ± 46 nm, which is consistent with the *z*-average diameter of 120 ± 27 nm reported by DLS. On the basis of TEM analysis, the larger standard deviation indicated by SAXS appears to be more reliable than that suggested by DLS. The mean membrane thickness was 5.5 nm, which indicates significant interdigitation of the structure-directing hydrophobic chains;^121^ similar observations were reported for thermoresponsive PHBA-based nano-objects.^46,58,59^ Finally, a relatively broad structure peak (q* = 0.024 Å) becomes visible at 75 °C, which suggests the presence of stacked lamellar sheets.^46^ This is consistent with TEM studies of the nano-objects that are formed at 75 °C (see Figure 5). For this SAXS pattern, the relation *d* = 2π /q* was used to estimate a mean intersheet separation distance of 26 nm.^46^

**Figure 6:**
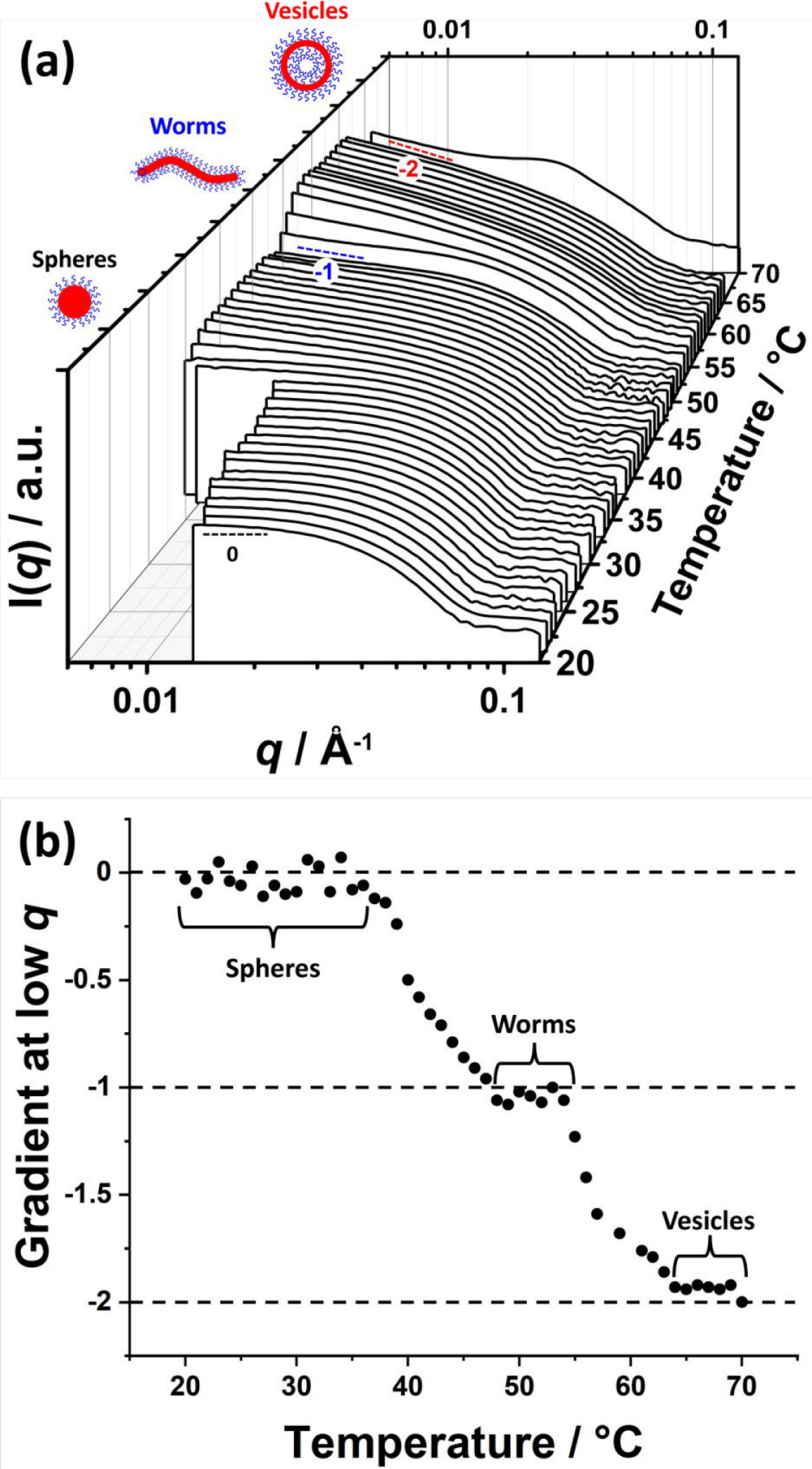
(a) SAXS patterns recorded between 20 and 70 °C for a 1.0% w/w aqueous dispersion of thermoresponsive PEG_45_–PHBMA_20_ nano-objects prepared at 10% w/w solids with a heating rate of 1 °C min^–1^. The characteristic low *q* gradients expected for spheres, worms, and vesicles (0, −1, and −2, respectively) are included as a guide for the eye (see the black, bluedashed lines indicate the characteristic and red dashed lines). In addition, there is a prominent structure factor in the final SAXS pattern recorded at 70 °C, which indicates interlamellar stacking. (b) Variation in the low *q* gradient (0.006 ≤ *q* ≤ 0.015 Å^–1^) for the SAXS patterns shown in (a) as a function of temperature. The horizontal dashed lines indicate the characteristic low *q* gradients (0, −1, and −2) which indicate the presence of spheres, worms and vesicles, respectively.

A series of SAXS patterns recorded for a 1.0% w/w aqueous dispersion of PEG_45_–PHBMA_20_ nano-objects on heating from 20 to 70 °C at 1 °C min^–1^ (Figure 6a) clearly demonstrate morphological transformation from spheres to worms to vesicles and, finally, to lamellae. In each case, the gradient of the scattering intensity in the low *q* region is characteristic of the predominant copolymer morphology, as shown in Figure 6b.^122^ The low *q* gradient is close to zero from 20 to 40 °C, suggesting the presence of spheres over this temperature range. At around 50 °C, the low *q* gradient tends toward −1, indicating the formation of highly anisotropic worms. At around 65 °C, the low *q* gradient is close to −2, which is characteristic of bilayer (or vesicle) formation. At 70 °C, the broad structure factor observed at around 0.024 Å indicates the presence of stacked lamellae sheets.^46^ Clearly, these SAXS data are consistent with the copolymer morphologies observed by TEM and rheology.

Moreover, SAXS analysis also indicated that the water volume fraction associated with the core-forming PHBMA block, *x*_sol_, remained constant at around 0.001 on heating from 20 to 50 °C (see Table 1). However, *x*_sol_ increased significantly to 0.18 on heating to 65 °C. A subtle increase in the (partial) degree of hydration of the core-forming block has been shown to be responsible for thermally-induced morphological transitions exhibited by diblock copolymer nano-objects prepared via RAFT aqueous dispersion polymerization.^39,46^ For example, Deane et al. used SAXS to calculate that *x*_sol_ for the PHBA core-forming block increased from 0.10 to 0.68 on heating PEG_113_–PHBA_260_ nano-objects from 10 to 50 °C.^59^ These *x*_sol_ values are consistent with those calculated for PEG_45_–PHBMA_20_ nano-objects in this study, particularly given that PHBMA is significantly more hydrophobic than PHBA.^35^ Thus, PHBMA is expected to become somewhat less hydrated than PHBA on heating. Nevertheless, the increase in (partial) hydration of the former block is clearly sufficient to cause PEG_45_–PHBMA_20_ nano-objects to undergo morphological transitions at elevated temperatures.

It is well-known that thermoresponsive PHPMA-based worms undergo a worm-to-sphere transition on *cooling* because of (partial) hydration of the HPMA repeat units that lie close to the block junction.^39,45^ This LCST-like behavior has been described as *surface* plasticization.^3^ In contrast, PHBA-based nano-objects exhibit UCST-like behavior: in this case, an increase in (partial) hydration of the hydrophobic block occurs on *heating*, which corresponds to *uniform* plasticization.^46,109^ Accordingly, variable temperature ^1^H NMR spectroscopy studies were conducted to elucidate the molecular mechanism responsible for the thermoresponsive behavior observed for PEG_45_–PHBMA_20_ nano-objects.

**Figure 7:**
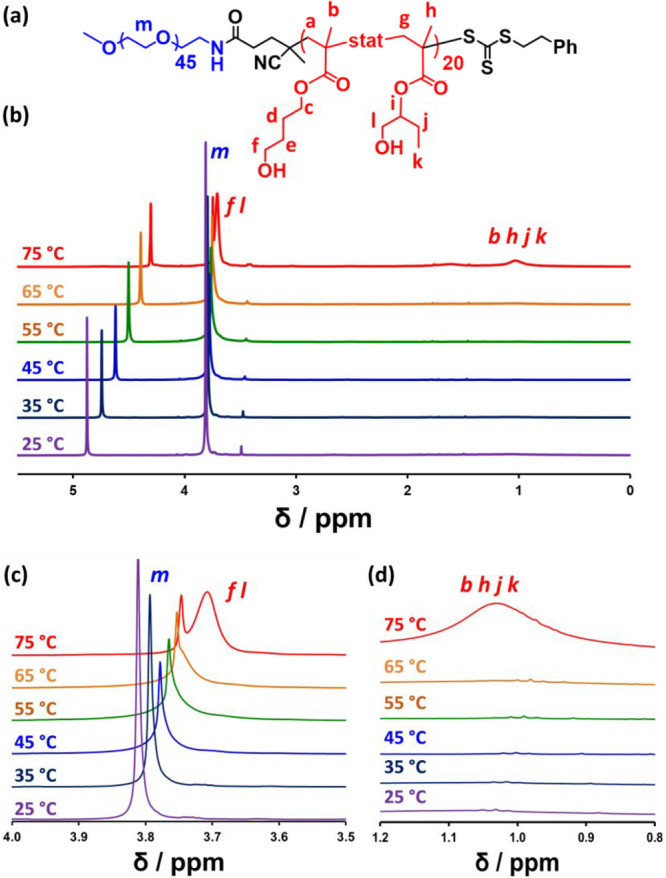
Variable temperature ^1^H NMR studies of thermoresponsive PEG_45_–PHBMA_20_ diblock copolymer nano-objects. (a) Chemical structure of the PEG_45_–PHBMA_20_ diblock copolymer showing the two types of HBMA repeat units. (b) Normalized (relative to a pyridine external standard) ^1^H NMR spectra recorded from 25 to 75 °C for a 10% w/w aqueous dispersion of PEG_45_–PHBMA_20_ nano-objects prepared in D_2_O. (c) Overlaid partial spectra recorded between 25 °C (purple) and 75 °C (red) for the oxymethylene protons (*m*) assigned to the PEG_45_ chains. This signal clearly becomes broader and weaker at higher temperatures, suggesting that this steric stabilizer block becomes less hydrated. (d) Overlaid partial spectra recorded between 25 °C (purple) and 75 °C (red). The signal at around 0.95–1.15 ppm assigned to the methacrylic backbone protons (*b*, *h*, *j*, *k*) of the PHBMA chains becomes visible at higher temperatures, indicating partial solvation of this block. In addition, a broad signal (*f*, *l*) at around 3.70–3.75 ppm is observed as a shoulder on the oxymethylene proton signal (*m*) assigned to the PEG_45_ chains at and above 65 °C, see panel (c). This new signal is assigned to the HO–CH_2_– protons on the HBMA repeat units.

Variable temperature ^1^H NMR spectroscopy studies were conducted between 20 and 75 °C on a 10% w/w aqueous dispersion of PEG_45_–PHBMA_20_ nano-objects prepared in D_2_O. ^1^H NMR spectra normalized relative to an external standard (pyridine) are shown in Figure 7, along with two sets of partial spectra highlighting regions of particular interest. ^1^H NMR signals assigned to the PEG_45_ stabilizer chains become broader and less prominent at higher temperature, indicating a progressively lower degree of hydration for this water-soluble block. This is consistent with literature data for aqueous solutions of PEO homopolymers, which undergo phase separation at elevated temperatures.^123−125^ In contrast, ^1^H NMR signals assigned to the oxymethylene (*m*) and methacrylic backbone protons (*b*, *h*, *j*, *k*) of the structure-directing PHBMA chains at around 3.7 and 1.0 ppm respectively, become progressively more intense on heating (see Figures 7c and 7d). This indicates that this weakly hydrophobic block becomes more hydrated, particularly at higher temperatures. These observations are consistent with the SAXS studies, which indicate a significant increase in the solvent volume fraction, *x*_sol_, associated with the PHBMA chains between 55 and 65 °C (see Table 1). However, it is rather surprising that such spectral changes only become apparent above 65 °C, whereas the sphere-to-worm and worm-to-vesicle transitions occur at significantly lower temperatures. Unfortunately, such spectral changes cannot be easily quantified because of overlap between the PEG and PHBMA signals at 3.7–3.8 ppm. Nevertheless, these ^1^H NMR studies indicate that a significant reduction in the degree of hydration of the PEG stabilizer chains occurs on heating. Indeed, the spectra shown in Figure 7 suggest that the sphere-to-worm and worm-to-vesicle transitions appear to be mainly driven by (partial) dehydration of the PEG stabilizer block, whereas the vesicle-to-lamellae transition is driven by an increase in the degree of (partial) hydration of the PHBMA block. We now seek to rationalize this hypothesis in terms of the fractional packing parameter, *P*, originally introduced by Israelachvili and co-workers to account for the micellization of small-molecule surfactants^56^ and more recently applied to the self-assembly of amphiphilic diblock copolymers.^57^

The packing parameter *P* is given by the following equation:
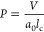
1For the PEG_45_–PHBMA_20_ diblock copolymer, *V* is the volume occupied by the hydrophobic PHBMA block, *a*_0_ is the optimal area occupied by the head-group (in this case, the PEG stabilizer), and *l*_c_ is the length of the PHBMA block.

**Figure 8:**
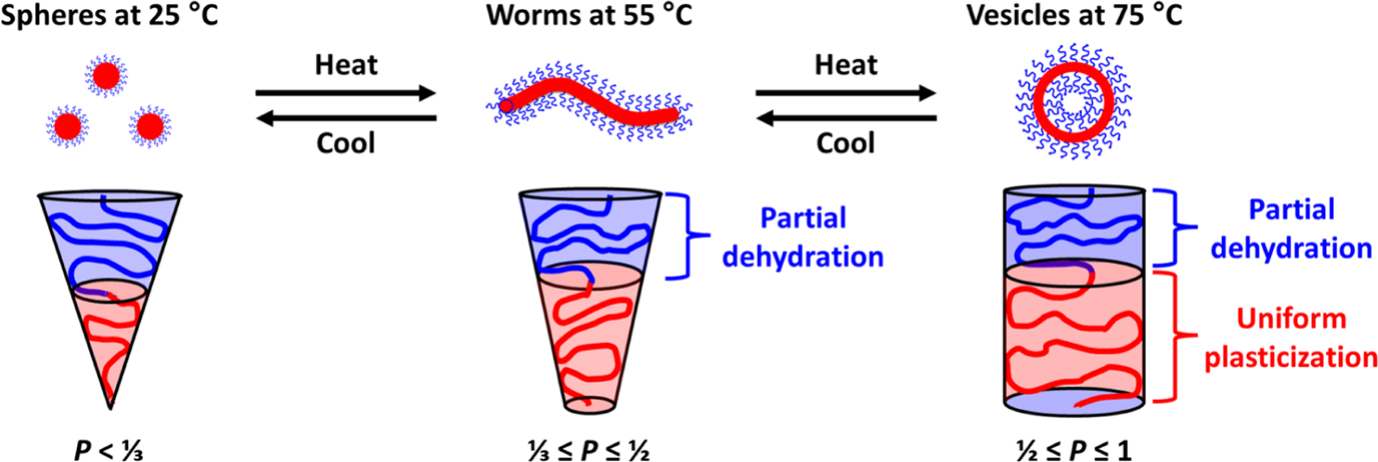
Schematic representation of the partial dehydration of the blue PEG stabilizer block at 55 °C and the uniform plasticization of the red core-forming PHBMA block at 65 °C that occur on heating an aqueous dispersion of PEG_45_–PHBMA_20_ spheres. These subtle changes account for the observed sphere-to-worm and worm-to-vesicle transitions, respectively.

It is well-known that aqueous solutions of PEG homopolymer exhibit LCST-type behavior.^126−130^ In principle, partial dehydration of the PEG stabilizer chains at higher temperatures should lead to an increase in the packing parameter *P*, which would account for the observed sphere-to-worm and worm-to-vesicle transitions (spheres are formed when *P* < 1/3 and worms are favored when 1/3 < *P* < 1/2). However, variable temperature SAXS and ^1^H NMR studies indicate that there is also a subtle increase in the (partial) degree of hydration of the structure-directing PHBMA block between 65 and 75 °C. If *surface* plasticization of the PHBMA block occurred at 75 °C, the HBMA residues near the block junction would become hydrated and *V* would decrease, which would lead to a concomitant *reduction* in *P*. However, this is not consistent with the experimental observations because this indicates that a vesicle-to-worm transition should occur. In contrast, *uniform* plasticization of the PHBMA block increases *V* and hence leads to the desired increase in *P*. Hence, *uniform* plasticization most likely accounts for the vesicle-to-lamellae transition exhibited by the PEG_45_–PHBMA_20_ diblock copolymer, as depicted in Figure 8. Similar UCST-like behavior has been reported for PHBA-based diblock copolymers.^46,58^ It is perhaps also worth noting that the likely further (partial) dehydration of the PEG stabilizer chains would also lead to an increase in the relative volume fraction of the hydrophobic PHBMA block.

**Figure 9:**
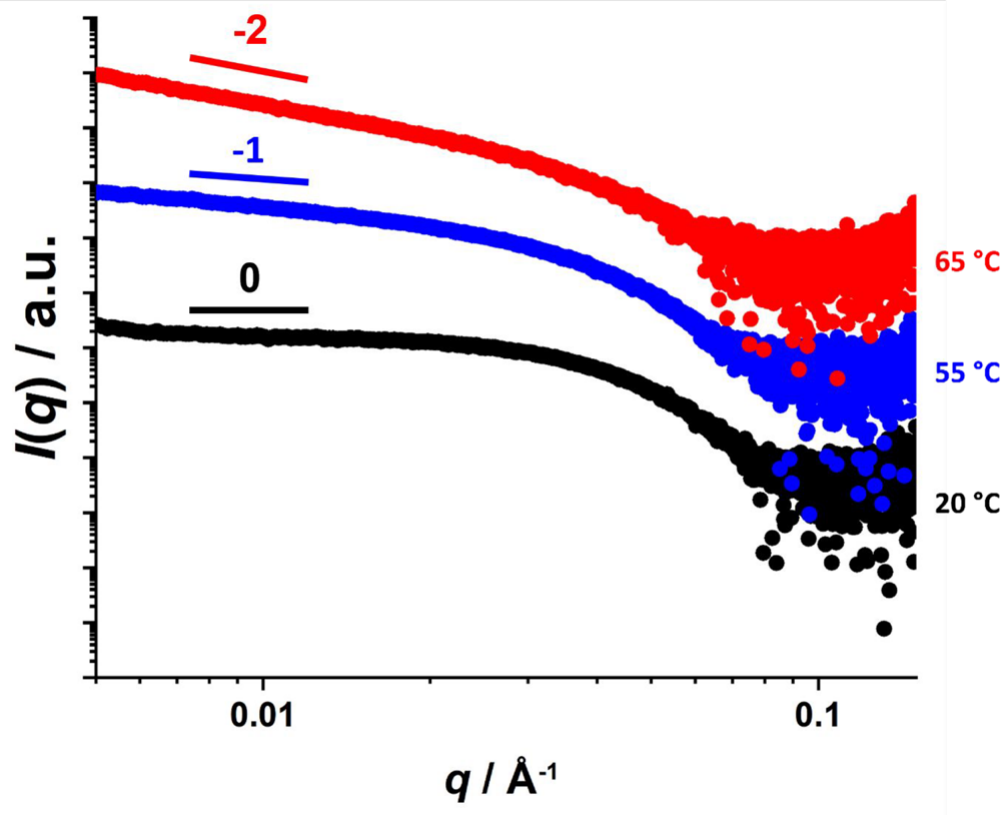
Representative double-logarithmic *I*(*q*) vs *q* SAXS patterns recorded for a 1.0% w/w aqueous dispersion of thermoresponsive PEG_45_–PHBMA_20_ nano-objects prepared at 30% w/w solids at 20 °C (black), 55 °C (blue) and 65 °C (red). The characteristic low *q* gradients expected for spheres, worms and vesicles (0, −1, and −2, respectively) are included as a guide for the eye.

As far as we are aware, this study constitutes the first example of amphiphilic diblock copolymer nano-objects prepared via RAFT aqueous emulsion polymerization that display thermoresponsive behavior. However, HBMA has a relatively high aqueous solubility of 25 g dm^–3^ at 50 °C. Thus, when targeting PEG_45_–PHBMA_20_ nano-objects at 10% w/w solids, a significant minority (∼42%) of this monomer is soluble within the aqueous continuous phase at this reaction temperature. Arguably, such aqueous PISA formulations lie somewhere between an aqueous emulsion polymerization and an aqueous dispersion polymerization. To increase the proportion of water-immiscible HBMA monomer, the synthesis of PEG_45_–PHBMA_20_ nano-objects was repeated targeting 30% w/w solids. Under such conditions, approximately 86% of the HBMA is water-immiscible at 50 °C. Gratifyingly, the resulting PEG_45_–PHBMA_20_ spheres displayed similar thermoreversible behavior as those prepared at 10% w/w solids. More specifically, variable temperature SAXS experiments confirmed that heating such nanoparticles from 20 to 55 °C led to the formation of worms. Moreover, further heating to 65 °C led to the formation of vesicles (see Figure 9).

## Conclusions

Amphiphilic PEG_45_–PHBMA_20_ diblock copolymer nano-objects have been prepared by chain-extending a water-soluble PEG_45_-TTC precursor via RAFT aqueous emulsion polymerization of HBMA at 50 °C. More than 99% conversion was achieved within 1 h, and efficient extension of the PEG_45_-TTC precursor with HBMA was confirmed by GPC analysis. Heating a transparent free-flowing 10% w/w dispersion of PEG_45_–PHBMA_20_ nano-objects up to 50 °C resulted in a sol–gel transition, which indicated the formation of a worm phase. At 65 °C, this dispersion became opaque and free-flowing, which suggested a worm-to-vesicle transition. After drying a 0.1% w/w aqueous dispersion of PEG_45_–PHBMA_20_ nano-objects at 25, 55, 65 or 75 °C, TEM studies confirmed the presence of spheres, worms, vesicles or lamellae, respectively. Variable temperature DLS studies confirmed that these morphological transitions are thermoreversible at copolymer concentrations as low as 0.1% w/w. Oscillatory rheology studies of a 10% w/w aqueous dispersion of these PEG_45_–PHBMA_20_ nano-objects as a function of temperature indicated a critical gelation temperature (CGT) of ∼52 °C, and the complex viscosity |η*| attained its maximum value at 58 °C. Furthermore, the morphological transitions for these shape-shifting nano-objects exhibited minimal hysteresis. As expected, SIPLI studies confirmed the presence of isotropic nano-objects at ambient temperature, the formation of highly anisotropic nano-objects at around 58 °C, and the re-emergence of isotropic nano-objects on further heating. Moreover, this technique indicated that anisotropic character is regained at 75 °C, as expected for a lamellar phase. Variable temperature ^1^H NMR spectroscopy studies indicated that the sphere-to-worm and worm-to-vesicle transitions are driven by (partial) dehydration of the PEG stabilizer chains rather than by (partial) hydration of the hydrophobic PHBMA chains. However, a greater (partial) degree of hydration of the PHBMA chains was observed above 65 °C, which is consistent with the higher solvent volume fraction within the corresponding nano-objects indicated by SAXS analysis. Consideration of the fractional packing parameter suggests that the associated vesicle-to-lamellae transition must occur by a uniform plasticization mechanism, which is similar to the thermoresponsive behavior reported for PHBA-based nano-objects.^46,58,59^ As far as we are aware, this is the first example of any thermoresponsive diblock copolymer nano-objects prepared via RAFT aqueous emulsion polymerization. Moreover, this particular PEG_45_–PHBMA_20_ diblock copolymer exhibits three thermoreversible transitions in aqueous solution. No doubt such behavior is related to the relatively short DP targeted for the hydrophobic PHBMA block. Moreover, the aqueous solubility of HBMA is relatively high, which suggests that the PHBMA block cannot be strongly hydrophobic. Clearly, further studies are warranted to examine whether similar thermoresponsive behavior can be achieved when targeting higher DPs for the PEG stabilizer and PHBMA blocks, respectively. However, it seems highly unlikely that such thermoresponsive behavior could ever be observed for more hydrophobic water-immiscible monomers such as styrene or benzyl methacrylate.

## Supporting Information Available

^1^H NMR spectrum recorded for PEG_45_–PHBMA_20_ diblock copolymer; DMF GPC curves recorded for both the PEG_45_-TTC precursor and PEG_45_–PHBMA_20_ diblock copolymer; variable-temperature rheology data obtained for a 10% w/w aqueous dispersion of PEG_45_–PHBMA_20_; SIPLI obtained for a 10% w/w aqueous dispersion of PEG_45_–PHBMA_20_ at 25, 60, 63, and 70 °C; schematic cartoon showing the difference in mean core cross-sectional radius for a sphere and cylinder of the same core volume; plot of SAXS patterns for a 1.0% w/w aqueous dispersion of PEG_45_–PHBMA_20_ nano-objects prepared at 10% w/w recorded between 20 and 70 °C; SAXS patterns for a 1.0% w/w aqueous dispersion of PEG_45_–PHBMA_20_ nano-objects prepared at 30% w/w recorded at 20, 55, and 65 °C; full experimental details of all syntheses and characterization methods; details of the three scattering models used for SAXS analysis (PDF)
