# Supplementary material for: Synthesis of Thermoresponsive Diblock Copolymer Nano-Objects via RAFT Aqueous Emulsion Polymerization of Hydroxybutyl Methacrylate
Source: Macromolecules. 2022 Apr 17;55(8):3051–62. doi: 10.1021/acs.macromol.2c00379 (PMC9047412; doi:10.1021/acs.macromol.2c00379)
Supplement: Supplementary file 1 — ma2c00379_si_001.pdf [file ma2c00379_si_001.pdf]

For Internal Use Only

Supporting Information for:

Synthesis of Thermoresponsive Diblock Copolymer Nano-Objects via RAFT Aqueous Emulsion Polymerisation of Hydroxybutyl Methacrylate

Saul J. Hunter[a], Nicholas J. W. Penfold[a], Elizabeth R. Jones,[b] Thomas Zinn,[c] Oleksandr O. Mykhaylyk[a] and Steven P. Armes[a,]*

a. Dainton Building, Department of Chemistry, University of Sheffield, Brook Hill, Sheffield, Yorkshire, S3 7HF, UK.

b.DSM Biomedical, Urmonderbaan 22, 6167RD Geleen, The Netherlands.

c. ESRF - The European Synchrotron, 38043 Grenoble, France

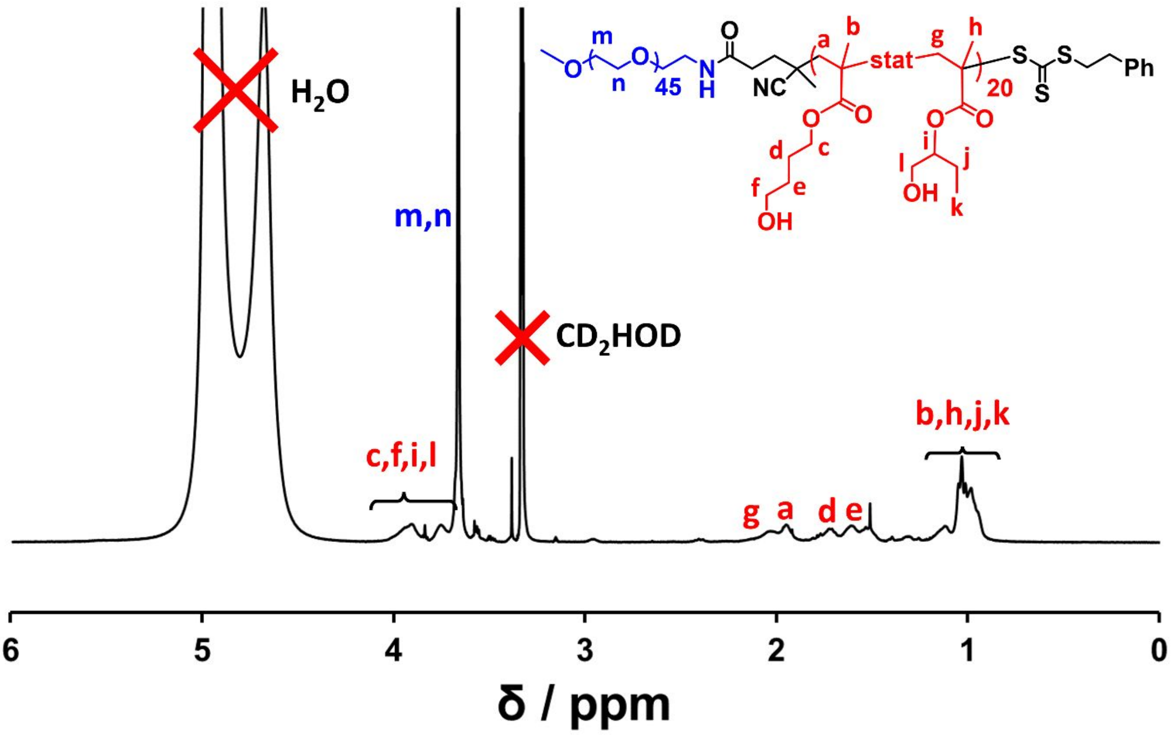

Figure S1. Assigned ^{1}H NMR spectrum (CD_3OD) recorded for a PEG_{45}-$PHBMA_{20}$ diblock copolymer obtained *via* RAFT aqueous emulsion polymerization of HBMA after more than 99% conversion was obtained within 60 min at 50°C. Both forms of the isomeric HBMA repeat units (which are present in a 1:1 molar ratio) are depicted in the chemical structure.

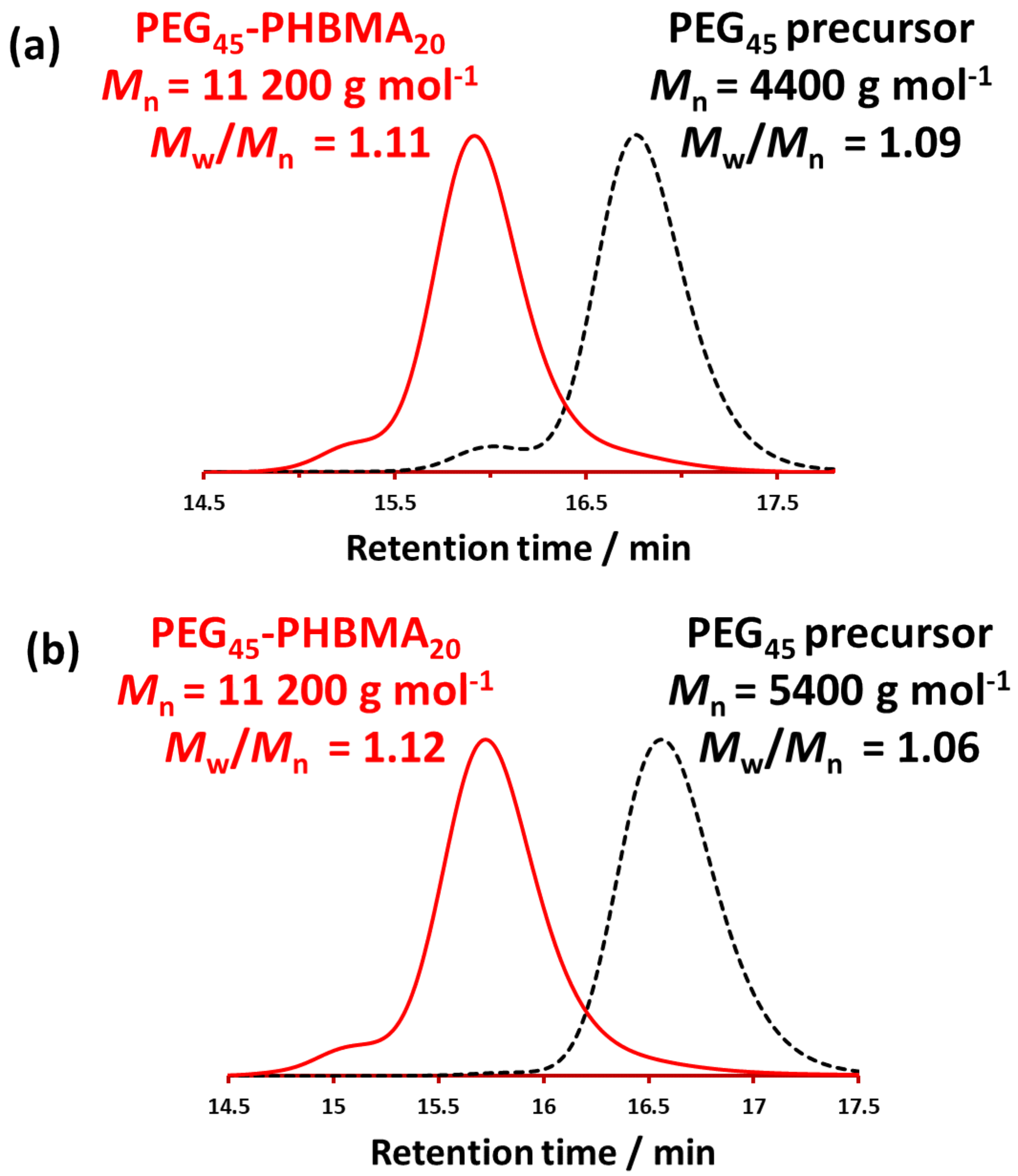

Figure S2. Overlaid DMF GPC curves recorded for a PEG_{45}-TTC precursor (black trace) and the corresponding PEG_{45}-$PHBMA_{20}$ diblock copolymer (red trace) prepared by RAFT aqueous emulsion polymerization of HBMA (conditions: 10% w/w, 50 °C, 1 h) using either (a) a refractive detector or (b) a UV detector (λ = 305 nm). Molecular weight data are expressed relative to a series of eleven near-monodisperse poly(methyl methacrylate) calibration standards.

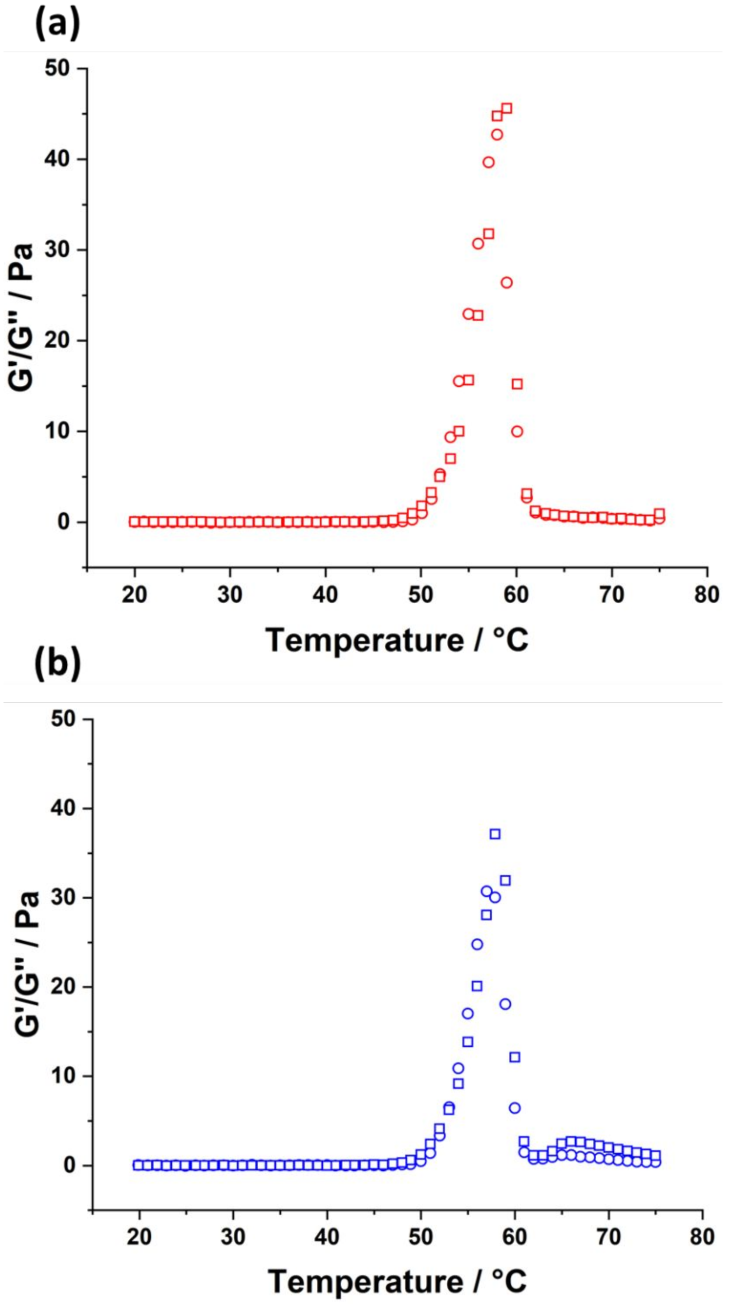

Figure S3. Storage (G'; circles) and loss (G''; squares) moduli recorded (a) on heating from 20 °C to 75 °C and (b) on cooling from 75 °C to 20 °C for a 10% w/w aqueous dispersion of PEG_{45}-$PHBMA_{20}$ nano-objects at an applied strain of 1.0% and an angular frequency of 1.0 rad s^{-1}. This dispersion was equilibrated at 20 °C for 10 min prior to a thermal cycle conducted at a heating/cooling rate of 1 °C min^{-1}.

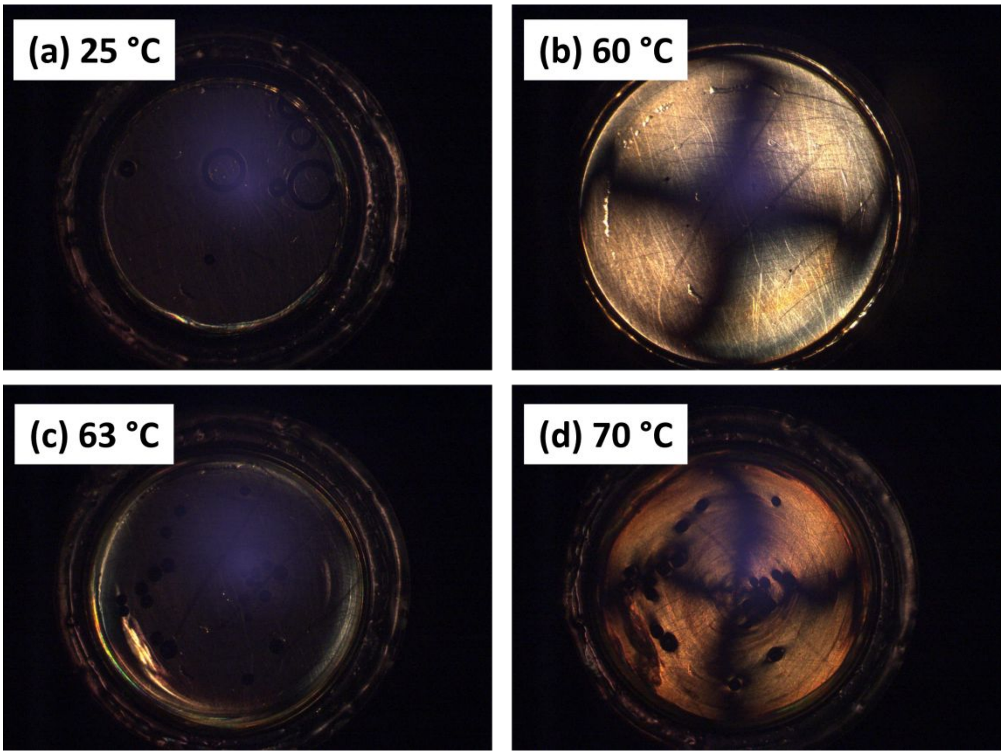

Figure S4. Shear-induced polarized light images (SIPLI) obtained for a 10% w/w aqueous dispersion of PEG_{45}-$PHBMA_{20}$ nano-objects at an applied maximum shear rate of 1.0 s^{-1} during a temperature ramp experiment conducted at a heating rate of 1.0 °C min^{-1}. The initial dark image recorded at 25 °C is consistent with the presence of isotropic spheres. In contrast, the highly anisotropic worms formed at 60 °C exhibit a characteristic Maltese cross owing to birefringence caused by their alignment in the direction of shear flow. This distinctive feature disappears at 63 °C, indicating the formation of isotropic vesicles. A second, weaker Maltese cross is observed at 70 °C, which is consistent with the presence of anisotropic lamellae.

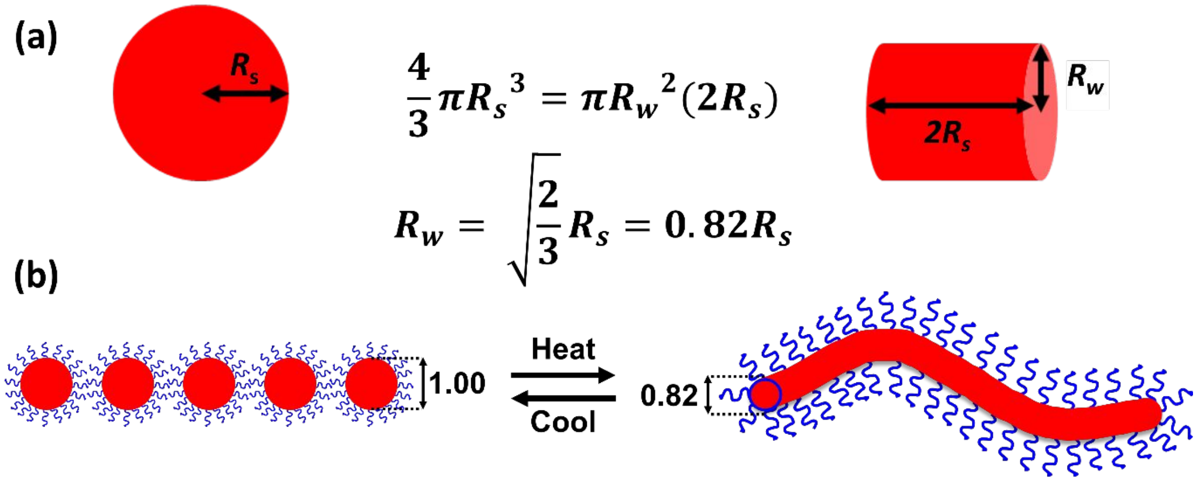

Figure S5. (a) Mean cross-sectional radius for a sphere and a cylinder of the same volume. (b) Reduction in mean cross-sectional radius associated with a sphere-to-worm transition while maintaining a constant core volume.[1]

Experimental

Materials

Hydroxybutyl methacrylate (HBMA; 94% purity; comprising a 1:1 mixture of 4-hydroxybutyl methacrylate and 2-hydroxybutyl methacrylate**)** was purchased from Sigma-Aldrich (Gillingham, UK) and used as received. A trithiocarbonate-based poly(ethylene glycol) (PEG_{45}-TTC, where the subscript refers to the mean degree of polymerization, or DP) precursor was prepared and purified as reported elsewhere.[2] 2,2′-Azobis[2-(2-imidazolin-2-yl)propane] dihydrochloride (VA-044; ≥ 97%) was purchased from Strem Chemicals Ltd. (Cambridge, UK) and used as received. CD_3OD was purchased from Goss Scientific Instruments Ltd. (Cheshire, UK). All other solvents were purchased from Fisher Scientific (Loughborough, UK) and were used as received. Deionized water was used for all experiments.

Synthesis of PEG_{45}-$PHBMA_{20}$ Diblock Copolymer Nano-Objects *via* RAFT Aqueous Emulsion Polymerization of HBMA

A 14 mL glass vial was charged with PEG_{45}-TTC precursor (0.40 g, 157 µmol), HBMA monomer (0.496 g, 3.13 mmol; target DP = 20), VA-044 initiator (10.1 mg, 31.3 µmol; PEG_{45}-TTC/VA-044 molar ratio = 5.0), and deionized water (8.17 g, 10% w/w solids). This reaction solution was purged with N_2 gas for 30 min at 20 °C prior to immersing the vial into an oil bath set at 50 °C. After 1 h, the HBMA polymerization was quenched by exposing the reaction mixture to air while cooling the vial to ambient temperature. A HBMA conversion of more than 99% was determined *via* ^{1}H NMR spectroscopy (integrated residual vinyl monomer signals at 6.18 ppm were compared to the methacrylic backbone signals at 0 − 2.50 ppm). THF GPC studies indicated an M_n of 5 900 g mol^{-1} and an M_w/M_n of 1.11 for the as-synthesized PEG_{45}-$PHBMA_{20}$ diblock copolymer.

Characterization

^{1}H NMR spectroscopy

Spectra were recorded in CD_3OD at 20 °C using a Bruker Avance III HD 400 MHz spectrometer with 64 scans being averaged per spectrum. Variable temperature ^{1}H NMR spectra were recorded using a 500 MHz Bruker Avance-500 spectrometer. In this case, an outer NMR tube contained a 10% w/w aqueous dispersion of PEG_{45}-$PHBMA_{20}$ nano-objects prepared in D_2O and an inner capillary tube contained an external standard (pyridine) dissolved in $C_2D_2Cl_4$. Spectra were recorded from 25 °C to 75 °C at 5 °C intervals with 10 min being allowed for thermal equilibration at each temperature and 64 scans being averaged per spectrum.

Gel permeation chromatography (GPC)

Aqueous copolymer solutions were diluted to 0.50% w/w using HPLC-grade DMF eluent containing 10 mM LiBr with DMSO (1.0 % v/v) being used as a flow-rate marker. GPC studies were conducted at 60 °C using a flow rate of 1.0 mL min^{-1}. The GPC set-up comprised an Agilent 1260 Infinity series degasser and pump, an Agilent PL-gel guard column, two Agilent PL-gel Mixed-C columns, a refractive index detector and a UV detector set at a wavelength of 305 nm. Eleven near-monodisperse poly(methyl methacrylate) standards with M_p values ranging from 2 380 g mol^{-1} to 2 200 000 g mol^{-1} were used for calibration.

Dynamic light scattering (DLS)

The hydrodynamic *z*-average diameter (D_z) and polydispersity index (PDI) were determined by DLS *via* the cumulants method using a Malvern Zetasizer NanoZS instrument. All measurements were performed on 0.10% w/w copolymer dispersions (prepared by dilution using deionized water) using disposable plastic cuvettes. All data were averaged over three consecutive runs. The 'sphere-equivalent' *z*-average diameter of diblock copolymer nano-objects was determined using the Stokes-Einstein equation, which assumes perfectly monodisperse, non-interacting spheres. For variable temperature DLS studies, 1.0 mL of a 0.10% w/w aqueous copolymer dispersion in a glass cuvette was heated from 20 °C to 75 °C at 1 °C intervals with 5 min being allowed for thermal equilibration at each temperature, followed by cooling from 75 °C to 20 °C using the same protocol. The hydrodynamic *z*-average diameter was determined at each temperature by averaging data over three consecutive runs.

Transmission electron microscopy (TEM)

Copper/palladium TEM grids (Agar Scientific, UK) were coated in-house to yield a thin film of amorphous carbon and then subjected to a plasma glow discharge for 30 s. One 10 μL droplet of each 0.10% w/w aqueous copolymer dispersion was placed in turn on a freshly-treated grid for 1 min and then carefully blotted with filter paper to remove excess solution. To ensure sufficient electron contrast, a 10 μL droplet of a 0.75% w/w aqueous uranyl formate solution was placed on the sample-loaded grid for 20 s and then blotted to remove excess stain. Each grid was then carefully dried using a vacuum hose. Imaging was performed using a FEI Tecnai Spirit 2 microscope operating at 80 kV and equipped with an Orius SC1000B camera.

Oscillatory Rheology

An AR-G2 rheometer (TA Instruments, Delaware, USA) equipped with a variable-temperature Peltier plate and a 40 mm 2° aluminum cone was used for all experiments. Temperature sweeps were conducted using a constant strain of 1.0% at an angular frequency of 1.0 rad s^{-1}. Prior to the temperature sweep, the 10% w/w PEG_{45}-$PHBMA_{20}$ aqueous copolymer dispersion was equilibrated at 20 °C for 5 min. Thermal cycles were conducted between 20 °C and 75 °C at 1 °C intervals.

Shear-induced polarized light imaging (SIPLI)

Polarized light images were recorded at various temperatures using a Physica MCR301 mechano-optical rheometer (Anton Paar, Graz, Austria) equipped with a SIPLI attachment and variable temperature Peltier (bottom plate and hood) heaters. A detailed description of this instrument can be found elsewhere.[3] A plate–plate geometry consisting of a 25 mm polished steel plate fixture and a fused quartz bottom plate with a fixed gap of 1.0 mm was used for these experiments. The angle between the polarizer and analyser was 90° and polarized light images were recorded under shear using a color CCD camera (Lumenera Lu165c) from 20 °C to 75 °C at a ramp rate of 1 °C min^{-1} for an 10% w/w aqueous dispersion of PEG_{45}-$PHBMA_{20}$ nano-objects. A maximum (sample edge) shear rate of 1.0 s^{-1} was applied for 250 s in each case.

Small Angle X-ray scattering (SAXS)

SAXS experiments were conducted on 1.0% w/w aqueous dispersions of PEG_{45}-$PHBMA_{20}$ nano-objects at the ESRF (station ID02, Grenoble, France) using monochromatic X-ray radiation (λ = 0.0995 nm; q range = 0.002 to 0.15 Å^{-1}, where q is the length of the scattering vector and θ is one-half of the scattering angle, such that $q = 4\pi \sin\theta/\lambda$) and a Eiger2 4M two-dimensional detector (Dectris, Switzerland). A glass capillary of 1.4 mm diameter was used as a sample holder and the temperature was controlled using a heating/cooling capillary holding stage (Linkam Scientific Instruments Ltd., Tadworth, UK). Scattering patterns were recorded from 20 °C to 75 °C at a heating rate of 1 °C min^{-1}. Scattering data were reduced using standard routines provided by the beamline and were further analyzed using Irena SAS macro for Igor Pro.[4]

Structural Models for Small-Angle X-ray Scattering Analysis

In general, the intensity of X-rays scattered by a dispersion of nanoparticles [usually represented by the scattering cross section per unit sample volume, $\frac{d\Sigma}{d\Omega}(q)$] can be expressed as:

$$\frac{d\Sigma}{d\Omega}(q) = \sum_{i=1}^{n} S_i(q) N_i \int_0^{\infty} \dots \int_0^{\infty} F_i(q, r_{1i}, \dots, r_{ki})^2 \Psi_i(r_{1i}, \dots, r_{ki}) dr_{1i} \dots dr_{ki} \qquad \textbf{S1}$$

where n is the number of different populations of particles in the dispersion, N_i is the number density of scattering particles of ith population, $F_i(q, r_{1i}, \dots, r_{ki})$ is the form factor that describes the particle morphology of the ith population using k_i number of parameters (including contrast and volume parameters of the particles), $\Psi_i(r_{1i}, \dots, r_{ki})$is the probability distribution function of parameters describing scattering particle model corresponding to the ith population, and S_i(q) is the structure factor arising from interparticle interactions.

Spherical Micelle Model

If a dispersion of particles is composed of a single population of spherical micelles, the spherical micelle form factor for eq S1 can be given by:[5]

$$F_{\mathrm{s\_mic}}(q) = N_{\mathrm{s}}^2\beta_{\mathrm{s}}^2A_{\mathrm{s}}^2(q,r_{\mathrm{s}}) + N_{\mathrm{s}}\beta_{\mathrm{c}}^2F_{\mathrm{c}}(q,R_{\mathrm{g}}) + N_{\mathrm{s}}(N_{\mathrm{s}}-1)\beta_{\mathrm{c}}^2A_{\mathrm{c}}^2(q) + 2\ldots(q) \quad \mathbf{S2}$$

where r_{s} is the radius of the spherical micelle core and R_{g} is the radius of gyration of the coronal steric stabilizer block. The R_{g} values obtained for the coronal blocks from all data fits are comparable to the estimated theoretical value, which can be calculated from the contour length of the PEG_{45} block, $L_{\mathrm{PEG}} = 45 \times 0.37$ nm = 16.7 nm (where the projected contour length of 0.37 nm for a single ethylene glycol repeat unit is derived from the crystal structure for PEG homopolymer[6]). Assuming a Kuhn length of PEG, b_{PEG}, is 1 nm for PEG,[7] an approximate R_{g} of $(16.7 \times 1/6)^{0.5} = 1.7$ nm $[R_{\mathrm{g}} = (b_{\mathrm{PEG}}L_{\mathrm{PEG}}/6)^{0.5}]$ was calculated for PEG_{45} chains.

The X-ray scattering length contrasts for the core and corona blocks are given by $\beta_{\mathrm{s}} = V_{\mathrm{s}}(\xi_{\mathrm{s}} - \xi_{\mathrm{sol}})$ and $\beta_{\mathrm{c}} = V_{\mathrm{c}}(\xi_{\mathrm{c}} - \xi_{\mathrm{sol}})$, respectively. Here, ξ_{s}, ξ_{c} and ξ_{sol} are the X-ray scattering length densities for the core block (ξ_{PHBMA}= 10.61 x 10^{10} cm^{-2}), corona block (ξ_{PEG}= 10.85 x 10^{10} cm^{-2}) and solvent (ξ_{water}= 9.42 x 10^{10} cm^{-2}), respectively and V_{s} and V_{c} are the volumes of the core-forming block (V_{PHBMA}) and the corona block (V_{PEG}), respectively. These volumes were calculated using $V = \frac{M_{\mathrm{n,\,pol}}}{N_{\mathrm{A}}\rho}$ where the mass density ρ of the core-forming block was taken to be $\rho_{\mathrm{PHBMA}} = 1.15$ g cm^{-3},[8] the density of stabilizer block was taken to be ρ_{PEG}= 0.97 g cm^{-3}, and the density of water was taken to be ρ_{water}= 1.00 g cm^{-3}.[9] $M_{\mathrm{n,\,pol}}$ corresponds to the number-average molecular weight of the diblock copolymer chains determined by ^{1}H NMR spectroscopy.

The sphere form factor amplitude is used for the amplitude of the core self-term:

$$A_{\mathrm{s}}(q,r_{\mathrm{s}}) = \Phi(qr_{\mathrm{s}})\exp\left(-\frac{q^2\sigma_{\mathrm{in}}^2}{2}\right) \quad \mathbf{S3}$$

where $\Phi(qr_{\mathrm{s}}) = \frac{3[\sin(qr_{\mathrm{s}}) - qr_{\mathrm{s}}\cos(qr_{\mathrm{s}})]}{(qr_{\mathrm{s}})^3}$. A sigmoidal interface between the two blocks was assumed for the spherical micelle form factor (Equation S3). This is described by the exponent term with a width

$\sigma_{\rm in}$ accounting for a decaying scattering length density at the micellar interface. This $\sigma_{\rm in}$ value was fixed at 0.25 nm during fitting.

The form factor amplitude of the spherical micelle corona is:

$$A_{\rm c}(q) = \frac{\int_{r_{\rm s}}^{r_{\rm s}+2s} \mu_{\rm c}(r)\frac{\sin(qr)}{qr}r^2 dr}{\int_{r_{\rm s}}^{r_{\rm s}+2s} \mu_{\rm c}(r) r^2 dr} exp\left(-\frac{q^2\sigma_{\rm in}^2}{2}\right) \quad \textbf{S4}$$

The radial profile of the corona, $\mu_{\rm c}(r)$, can be expressed by a linear combination of two cubic b-splines, with two fitting parameters s and a corresponding to the width of the profile and the weight coefficient, respectively. This information can be found elsewhere,[10-11] as can the approximate integrated form of eq S4.

The aggregation number, $N_{\rm s}$, of the spherical micelle is given by:

$$N_{\rm s}(r_s) = (1 - x_{\rm sol})\frac{\frac{4}{3}\pi {r_{\rm s}}^3}{V_{\rm s}} \quad \textbf{S5}$$

where $x_{\rm sol}$ is the volume fraction of solvent within the micelle core. A dispersity for one parameter ($r_{\rm s}$) is assumed for the micelle model, which is described by a Gaussian distribution. Thus, the dispersity function can be represented as:

$$\Psi(r_{\rm s}) = \frac{1}{\sqrt{2\pi {\sigma_{R_{\rm s}}}^2}} exp\left(-\frac{(r_{\rm s} - R_{\rm s})^2}{2{\sigma_{R_{\rm s}}}^2}\right) \quad \textbf{S6}$$

where $R_{\rm s}$ is the mean spherical micelle core radius and $\sigma_{R_{\rm s}}$ is its standard deviation. The number density per unit volume (eq S1) for the micelles is expressed as:

$$N = \frac{\varphi}{\int_0^\infty V(r_{\rm s})\Psi(r_{\rm s})dr_{\rm s}} \quad \textbf{S7}$$

where φ is the total volume fraction of copolymer in the spherical micelles and $V(r_{\rm s})$ is the total volume of copolymer in a spherical micelle $V(r_{\rm s}) = (V_{\rm s} + V_{\rm c})N_{\rm s}(r_{\rm s})$.

Worm-like Micelle Model

The worm-like micelle form factor for eq S1 could be given by:[5]

$$F_{\mathrm{w_{mic}}}(q,r_{\mathrm{w}}) = N_{\mathrm{w}}^{2}(r_{\mathrm{w}})\beta_{\mathrm{s}}^{2}F_{\mathrm{w}}(q,r_{\mathrm{w}}) + N_{\mathrm{w}}(r_{\mathrm{w}})\beta_{\mathrm{c}}^{2}F_{\mathrm{c}}(q,R_{\mathrm{g}}) + N_{\mathrm{w}}(r_{\mathrm{w}})[N_{\mathrm{w}}(r_{\mathrm{v}} \text{[illegible]} + 2N_{\mathrm{w}}^{2}(r_{\mathrm{w}})\beta_{\mathrm{s}}\beta_{\mathrm{c}}S_{\mathrm{sc}}(q,r_{\mathrm{w}}) \quad \mathbf{S8}$$

where all the parameters are the same as those in the spherical micelle model, unless stated otherwise. The self-correlation term for a worm core of cross-sectional radius r_{w} is given by:

$$F_{\mathrm{w}}(q,r_{\mathrm{w}}) = F_{\mathrm{worm}}(q,L_{\mathrm{w}},b_{\mathrm{w}})A_{\mathrm{csworm}}^{2}(q,r_{\mathrm{w}}) \quad \mathbf{S9}$$

where

$$A_{\mathrm{csworm}}^{2}(q,r_{\mathrm{w}}) = \left[2\frac{J_1(qr_{\mathrm{w}})}{qr_{\mathrm{w}}}\right]^{2} \quad \mathbf{S10}$$

J_1 is the first-order Bessel function of the first kind and a form factor $F_{\mathrm{worm}}(q,L_{\mathrm{w}},b_{\mathrm{w}})$ for self-avoiding semi-flexible chains represents the worm-like micelles, where b_{w} is the Kuhn length and L_{w} is the mean contour length of the hydrophobic (PHBMA) block. A complete expression for the chain form factor can be found elsewhere.[12]

The mean aggregation number of the worm-like micelle, $N_{\mathrm{w}}(r_{\mathrm{w}})$, is given by:

$$N_{\mathrm{w}}(r_{\mathrm{w}}) = (1 - x_{\mathrm{sol}})\frac{\pi r_{\mathrm{w}}^{2}L_{\mathrm{w}}}{V_{\mathrm{s}}} \quad \mathbf{S11}$$

where x_{sol} is the volume fraction of solvent within the worm-like micelle core. The possible presence of semi-spherical caps at both ends of each worm is neglected in this form factor.

A dispersity for a single parameter (r_{w}) is assumed for this worm-like micelle model, which is described by a Gaussian distribution. Thus, the polydispersity function can be represented as:

$$\Psi(r_{\mathrm{w}}) = \frac{1}{\sqrt{2\pi\sigma_{R_{\mathrm{w}}}^{2}}}exp\left(-\frac{(r_{\mathrm{w}} - R_{\mathrm{w}})^{2}}{2\sigma_{R_{\mathrm{w}}}^{2}}\right) \quad \mathbf{S12}$$

where R_{w} is the volume-average cross-sectional radius and $\sigma_{R_{\mathrm{w}}}$ is its standard deviation. The number density per unit volume (eq S1) for the worm-like micelles is expressed as:

$$N = \frac{\varphi}{\int_0^{\infty}V(r_{\mathrm{w}})\Psi(r_{\mathrm{w}})dr_{\mathrm{w}}} \quad \mathbf{S13}$$

where φ is the total volume fraction of copolymer in the worm-like micelles and $V(r_\mathrm{w})$ is the total volume of copolymer in a worm-like micelle $[V(r_\mathrm{w}) = (V_\mathrm{s} + V_\mathrm{c})N_\mathrm{w}(r_\mathrm{w})]$.

Vesicle Model

The vesicle form factor for eq S1 could be expressed as:[13]

$$F_\mathrm{ves}(q,r_\mathrm{v},T_\mathrm{v}) = N_\mathrm{v}^2(r_\mathrm{v},T_\mathrm{v})\beta_\mathrm{s}^2 A_\mathrm{m}^2(q,r_\mathrm{v},T_\mathrm{v}) + N_\mathrm{v}(r_\mathrm{v},T_\mathrm{v})\beta_\mathrm{c}^2 F_\mathrm{c}(q,R_\mathrm{g}) + N_\mathrm{v}(r_\mathrm{v},T \quad 1]\beta_\mathrm{c}^2 A_\mathrm{vc}^2(q) + 2N_\mathrm{v}^2(r_\mathrm{v},T_\mathrm{v})\beta_\mathrm{s}\beta_\mathrm{c} A_\mathrm{m}(q,r_\mathrm{v},T_\mathrm{v})A_\mathrm{vc}(q) \qquad \mathbf{S14}$$

where all the parameters are the same as those in the spherical micelle model, unless stated otherwise.

The amplitude of the membrane self-term is:

$$A_\mathrm{m}(q,r_\mathrm{v},T_\mathrm{v}) = \frac{V_\mathrm{out}\Phi(qR_\mathrm{out}) - V_\mathrm{in}\Phi(qR_\mathrm{in})}{V_\mathrm{out} - V_\mathrm{in}} exp\left(-\frac{q^2\sigma_\mathrm{in}^2}{2}\right) \qquad \mathbf{S15}$$

where $R_\mathrm{in} = r_\mathrm{v} - \frac{1}{2}T_\mathrm{v}$ is the inner radius of the membrane and $R_\mathrm{out} = r_\mathrm{v} + \frac{1}{2}T_\mathrm{v}$ is the outer radius of the membrane (r_v is the radius from the centre of the vesicle to the centre of the membrane and T_v is the membrane thickness), and $V_\mathrm{in} = \frac{4}{3}\pi R_\mathrm{in}^3$ and $V_\mathrm{out} = \frac{4}{3}\pi R_\mathrm{out}^3$. It should be noted that Equation S15 differs from that in the original study in which this scattering model was first introduced.[13] The mean vesicle aggregation number, $N_\mathrm{v}(r_\mathrm{v},T_\mathrm{v})$, is given by

$$N_\mathrm{v}(r_\mathrm{v},T_\mathrm{v}) = (1 - x_\mathrm{sol})\frac{V_\mathrm{out} - V_\mathrm{in}}{V_\mathrm{s}} \qquad \mathbf{S16}$$

where x_sol is the volume fraction of solvent within the vesicle membrane. Assuming that there is no penetration of the hydrophilic coronal blocks into the hydrophobic membrane, the amplitude of the vesicle corona self-term can be expressed as:

$$A_\mathrm{vc}(q) = \Psi(qR_\mathrm{g})\frac{1}{2}\left[\frac{sin[q(R_\mathrm{out} + R_\mathrm{g})]}{q(R_\mathrm{out} + R_\mathrm{g})} + \frac{\sin[q(R_\mathrm{in} - R_\mathrm{g})]}{q(R_\mathrm{in} - R_\mathrm{g})}\right] \qquad \mathbf{S17}$$

where the term outside the square brackets is the form factor amplitude of the coronal block of the copolymer chain such that:

$$\Psi(qR_{\mathrm{g}}) = \frac{1-\exp\left(-qR_{\mathrm{g}}\right)}{(qR_{\mathrm{g}})^2} \qquad \mathbf{S18}$$

For this vesicle model, it is assumed that two parameters exhibit finite dispersity: the radius from the centre of the vesicle to the centre of the membrane and the mean membrane thickness (denoted as r_{v} and $T_{\mathrm{v}},$ respectively). Each parameter is considered to have a Gaussian distribution of values, so the dispersity function can be expressed as:

$$\Psi(r_{\mathrm{v}},T_{\mathrm{v}}) = \frac{1}{\sqrt{2\pi\sigma_{R_{\mathrm{s}}}{}^2}} exp\left(-\frac{(r_{\mathrm{v}}-R_{\mathrm{m}})^2}{2\sigma_{R_{\mathrm{m}}}{}^2}\right)\frac{1}{\sqrt{2\pi\sigma_{T_{\mathrm{m}}}{}^2}} exp\left(-\frac{(T_{\mathrm{v}}-T_{\mathrm{m}})^2}{2\sigma_{T_{\mathrm{m}}}{}^2}\right) \qquad \mathbf{S19}$$

where R_{m} is the mean radius from the centre of the vesicle to the centre of the membrane and T_{m} is the mean membrane thickness. Here $\sigma_{R_{\mathrm{m}}}$ and $\sigma_{T_{\mathrm{m}}}$ are the corresponding standard deviations for R_{m} and T_{m}, respectively. The number density per unit volume (eq S1) for the vesicles is expressed as:

$$N = \frac{\varphi}{\int_0^\infty\int_0^\infty V(r_{\mathrm{v}},T_{\mathrm{v}})\Psi(r_{\mathrm{v}},T_{\mathrm{v}})dr_{\mathrm{v}}dT_{\mathrm{v}}} \qquad \mathbf{S20}$$

where φ is the total *volume fraction* of copolymer in the vesicles and $V(r_{\mathrm{v}},T_{\mathrm{v}})$ is the total *volume* of copolymer within a single vesicle $[V(r_{\mathrm{v}},T_{\mathrm{v}}) = (V_{\mathrm{s}}+V_{\mathrm{c}})N_{\mathrm{v}}(r_{\mathrm{v}},T_{\mathrm{v}})]$.

Programming tools within the Irena SAS Igor Pro macros[4] were used to implement the scattering models.

References

1. Beattie, D. L.; Mykhaylyk, O. O.; Ryan, A. J.; Armes, S. P., Rational synthesis of novel biocompatible thermoresponsive block copolymer worm gels. *Soft Matter* **2021,** *17*, 5602-5612.

2. Penfold, N. J. W.; Whatley, J. R.; Armes, S. P., Thermoreversible Block Copolymer Worm Gels Using Binary Mixtures of PEG Stabilizer Blocks. *Macromolecules* **2019,** *52*, 1653-1662.

3. Mykhaylyk, O. O.; Warren, N. J.; Parnell, A. J.; Pfeifer, G.; Laeuger, J., Applications of shear-induced polarized light imaging (SIPLI) technique for mechano-optical rheology of polymers and soft matter materials. *Journal of Polymer Science Part B: Polymer Physics* **2016,** *54*, 2151-2170.

4. Ilavsky, J.; Jemian, P. R., Irena: tool suite for modeling and analysis of small-angle scattering. *J. Appl. Crystallogr.* **2009,** *42*, 347-353.

5. Pedersen, J., Form factors of block copolymer micelles with spherical, ellipsoidal and cylindrical cores. *J. Appl. Crystallogr.* **2000,** *33*, 637-640.

6. Takahashi, Y.; Tadokoro, H., Structural Studies of Polyethers, (-(CH2)m-O-)n. X. Crystal Structure of Poly(ethylene oxide). *Macromolecules* **1973,** *6*, 672-675.

7. Fetters, L. J.; Lohse, D. J.; Richter, D.; Witten, T. A.; Zirkel, A., Connection between Polymer Molecular Weight, Density, Chain Dimensions, and Melt Viscoelastic Properties. *Macromolecules* **1994,** *27*, 4639-4647.

8. Cockram, A. A.; Neal, T. J.; Derry, M. J.; Mykhaylyk, O. O.; Williams, N. S. J.; Murray, M. W.; Emmett, S. N.; Armes, S. P., Effect of Monomer Solubility on the Evolution of Copolymer Morphology during Polymerization-Induced Self-Assembly in Aqueous Solution. *Macromolecules* **2017,** *50*, 796-802.

9. Derry, M. J.; Fielding, L. A.; Warren, N. J.; Mable, C. J.; Smith, A. J.; Mykhaylyk, O. O.; Armes, S. P., In situ small-angle X-ray scattering studies of sterically-stabilized diblock copolymer nanoparticles formed during polymerization-induced self-assembly in non-polar media. *Chem. Sci.* **2016,** *7*, 5078-5090.

10. Pedersen, J. S.; Gerstenberg, M. C., The structure of P85 Pluronic block copolymer micelles determined by small-angle neutron scattering. *Colloids Surf., A* **2003,** *213*, 175-187.

11. Pedersen, J. S.; Svaneborg, C.; Almdal, K.; Hamley, I. W.; Young, R. N., A Small-Angle Neutron and X-ray Contrast Variation Scattering Study of the Structure of Block Copolymer Micelles: Corona Shape and Excluded Volume Interactions. *Macromolecules* **2003,** *36*, 416-433.

12. Pedersen, J. S.; Schurtenberger, P., Scattering Functions of Semiflexible Polymers with and without Excluded Volume Effects. *Macromolecules* **1996,** *29*, 7602-7612.

13. Bang, J.; Jain, S.; Li, Z.; Lodge, T. P.; Pedersen, J. S.; Kesselman, E.; Talmon, Y., Sphere, Cylinder, and Vesicle Nanoaggregates in Poly(styrene-b-isoprene) Diblock Copolymer Solutions. *Macromolecules* **2006,** *39*, 1199-1208.
